# ORAC: The Method of Choice for Determining Antioxidant Capacity of Food Products?

**DOI:** 10.3390/ijms27114825

**Published:** 2026-05-27

**Authors:** Izabela Sadowska-Bartosz, Grzegorz Bartosz

**Affiliations:** Laboratory of Analytical Biochemistry, Institute of Food Technology and Nutrition, Faculty of Technology and Life Sciences, University of Rzeszow, 4 Aleksandra Zelwerowicza Street, 35-601 Rzeszow, Poland; gbartosz@ur.edu.pl

**Keywords:** alkoxyl radical, antioxidant activity, extraction, fluorescence, fluorescein, ORAC, peroxyl radical, Pyrogallol Red, total antioxidant capacity

## Abstract

The Oxygen Radical Absorbance Capacity (ORAC) assay is one of the most popular assays of antioxidant activity/capacity. It has several advantages over other common assays, including the use of an oxidant (peroxyl radicals) relevant in physiology and food storage and processing, as well as reactions in the physiological pH range and temperature. These advantages make ORAC the method of choice for the determination of antioxidant activity/capacity. This review presents the methodology and application of ORAC to the analysis of food products, various versions of the assay, including the lipophilic ORAC-related assays like the Hydroxyl Radical Absorbance Capacity (HORAC), Peroxynitrite Absorbance Capacity (NORAC), Superoxide Anion Absorbance Capacity (SORAC), and Singlet Oxygen Absorbance Capacity (SOAC); discusses the pros and cons, nd technical details affecting the reproducibility of ORAC. Examples of applications of the assay are given, including ORAC values [mol Trolox equivalent/mol, and mmol Trolox equivalents/kg or per L, respectively] for over 90 antioxidants and over 900 food products and medicinal plants.

## 1. Introduction

The idea of Total Antioxidant Capacity (TAC), understood as a parameter describing the sum of activities of all antioxidants present in a sample, reflecting interactions between them, derivable from a single assay [1], appeared attractive to many researchers [2,3,4,5]. Quite a range of methods has been used to estimate TAC, the most popular being the DPPH^•^ decolorization assay, the ABTS^•^ decolorization assay, the Ferric Reducing Antioxidant Power (FRAP) assay, the CUPric ion Reducing Antioxidant Capacity (CUPRAC) assay, the ferricyanide reduction assay, the Total Radical trapping Antioxidant Parameter (TRAP) assay and, last but not least, the Oxygen Radical Absorbance Capacity (ORAC) assay. According to the Google Scholar database, ORAC (about 53,500 hits) is the fifth most popular antioxidant assay, after DPPH^•^ decolorization, TRAP, ABTS^•^ decolorization and FRAP (about 414,000, 357,000, 150,000, and 101,800 hits, respectively). According to Pubmed, ORAC (2858 articles) is the fourth most popular antioxidant assay, after DPPH^•^ decolorization, ABTS^•^ decolorization, and FRAP (32,411, 14,185, and 9608 articles, respectively; data obtained searching the combination of terms “assay name” and “antioxidant” on 26 May 2026). Many other assays are also used [6,7,8,9].

All TAC assays have been criticized. One of the main points of this criticism concerns the use of non-physiological oxidants employed in most assays and the non-physiological reaction milieu of some assays. Indeed, the knowledge of the reactivity of oxidants with synthetic substrates such as ABTS^•^ or DPPH^•^ may be of limited physiological relevance. The CUPRAC assay is based on the reduction of Cu^2+^ ions [10], which occur in cells and body fluids, but in amounts too low to be a significant oxidant, and is practically absent in food products. Most proteins precipitate in methanol, which is used as the medium of the DPPH^•^ decolorization assay. The FRAP assay employs a pH of 3.6, which is far from physiological pH [11]. Moreover, the correlation between the results of different assays is often moderate. What is even more important, the mechanisms of various assays differ. ABTS^•^ and DPPH^•^ decolorization assays are based on the reductive neutralization of stable free radicals and are in principle single electron transfer (SET) assays, although the mechanism of hydrogen atom transfer (HAT) may contribute [2,3,4,5,6,7,8,9]. The FRAP and CUPRAC assays are based on the reduction of Fe^3+^ or Cu^2+^ ions, respectively, and are purely SET reactions. The reactivity of various compounds with the substrates may vary; e.g., thiols are weakly reactive in the FRAP assay, while steric hindrance may limit the reactivity of some compounds with the indicators [2,3,4,5,6,7,8,9,10,11].

In comparison with these assays, ORAC presents several important advantages. It measures the reactivity of antioxidants with physiologically relevant peroxyl radicals ROO^•^, which are also important in food storage and processing. The assay employs a physiological pH range so that antioxidants react with an overall charge and protonation state similar to that in the body. It is run at a physiological temperature and provides a continuous flux of radicals on a realistic time scale (more like actual reactions in situ). It can measure the activities of both hydrophilic and lipophilic antioxidants. It is based on the hydrogen transfer (HAT) mechanism, which is the main mechanism of action of biological antioxidants. All these aspects speak in favor of considering the ORAC assay as the method of choice for the determination of antioxidant activity of various compounds and antioxidant capacity of biological fluids and food products. This review is aimed at presenting the principles and limitations of the ORAC assay and its application in the field of food science. Principles of similar assays, measuring the reactivity of the examined material with other, physiologically and technologically important reactive oxygen species, are also briefly presented.

## 2. Principle of the ORAC Assay

The ORAC assay was originally developed by Glazer [12,13] and Ghiselli et al. [14] and then refined and applied to extensive analyses of hundreds of foods by Prior and his collaborators [15,16,17,18,19,20,21,22,23,24]. The assay is based on the inhibition of the oxidation of a fluorescent or colored substrate by radicals generated after decomposition of a thermolabile substrate. The most commonly used substrate is 2,2′-azobis(2-methylpropionamidine) (AAPH). This azo compound decomposes spontaneously at a rate increasing with temperature. The decomposition follows the first-order kinetics

(1) $${\left[AAPH\right]}_{t}={\left[AAPH\right]}_{t=0\;}\times e^{k_{d}\times t}$$

where *k_d_* is the decomposition rate constant, and *t* is the time. In pure water, the value of the decomposition constant was found to be 4.28 × 10^−7^ s^−1^ at 30 °C and 1.45 × 10^−6^ s^−1^ at 37 °C, and the activation energy for decomposition was equal to 137 kJ/mol [25]. The decomposition rate depends on pH, weekly in the pH range of 3–7, but sharply increases with pH rising above 7 [26].

Decomposition of AAPH leads to the formation of two carbon-centered alkyl radicals (R^•^) and nitrogen gas (N_2_). The radicals may either rapidly recombine in a termination reaction or react with oxygen, forming peroxyl radicals. The rate of generation of peroxyl radicals, denoted as R_i_, is a critical factor in this reaction. At 37 °C and pH 7.4, the value of R_i_ is about 1.36 × 10^−6^ × [AAPH] s^−1^ if AAPH concentration is expressed in moles/L [27]. Thus, for 10 mM AAPH, the rate of generation of peroxyl radicals is 1.36 × 10^−8^ M radicals per second. The half-life of AAPH under these conditions is about 175 h, so R_i_ remains practically constant during a several-hour-long experiment.

**Figure 1:**
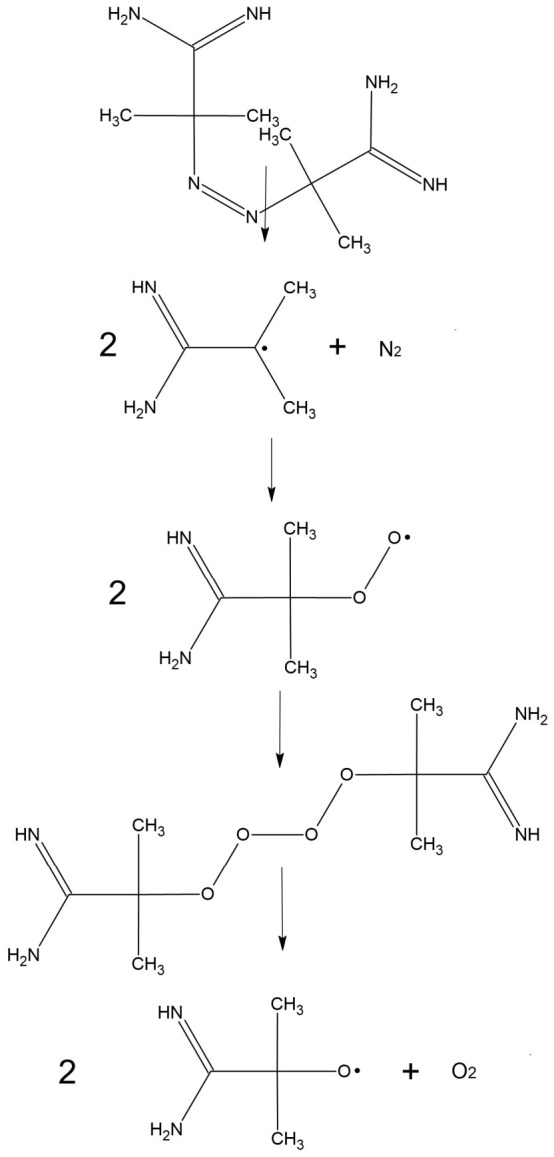
Scheme of AAPH decomposition.

In the absence of antioxidants, peroxyl radicals (ROO^•^) generated from AAPH primarily undergo self-reaction, leading to the formation of an unstable tetroxide intermediate (ROOOOR), which decomposes, forming alkoxyl radicals (RO^•^) and oxygen (O_2_) (Figure 1). These RO^•^ radicals are initially confined within the solvent cage, where they predominantly recombine to form non-radical products. A minority escapes into the solution to participate in further reactions [28,29].

Apart from AAPH, peroxyl radicals may be generated by lipophilic azo compounds such as 2,2′-azobis [2-(2-imidazolin-2-yl)propane] (AIPH), 2,2′-azobis(2,4-dimethylvaleronitrile) (AMVN), or 2,2′-azobis(4-methoxy-2,4-dimethylvaleronitrile) (Meo-AMVN) [30,31,32,33].

Initially, phycoerythrins were used as the fluorescent substrate. These proteins function as light-harvesting components in cyanobacteria and red algae. The fluorescence quantum yield of these proteins is >0.9 and provides the basis for sensitive measurements of the proteins’ physical and chemical integrity. *β*-Phycoerythrin (*β*-PE) used mainly in these studies, was isolated from *Porphyridium cruentum* [15]. A similar protein, *β*-phycocyanin (β-PC), often found in cyanobacteria, was also employed instead of *β*-phycoerythrin [34]. However, the use of β-PE in antioxidant assays has shortcomings: (i) *β*-PE has lot-to-lot variability in reactivity to peroxyl radicals, leading to inconsistency in assay results; (ii) *β*-PE becomes photobleached after exposure to excitation light; and (iii) polyphenols, particularly proanthocyanidins, non-specifically bind to *β*-PE. The latter factors may lower the results of ORAC measurements. In a more recent and most widely used version of the ORAC assay, β-PE is replaced by fluorescein (3′,6′-dihydroxy-spiro[isobenzofuran-1[3*H*],9′[9*H*]-xanthen]-3-one), which also has a high fluorescence quantum yield (>0.9 in the dianion form [35,36]) but is cheaper, more stable, and less reactive [19,20,37,38].

**Figure 2:**
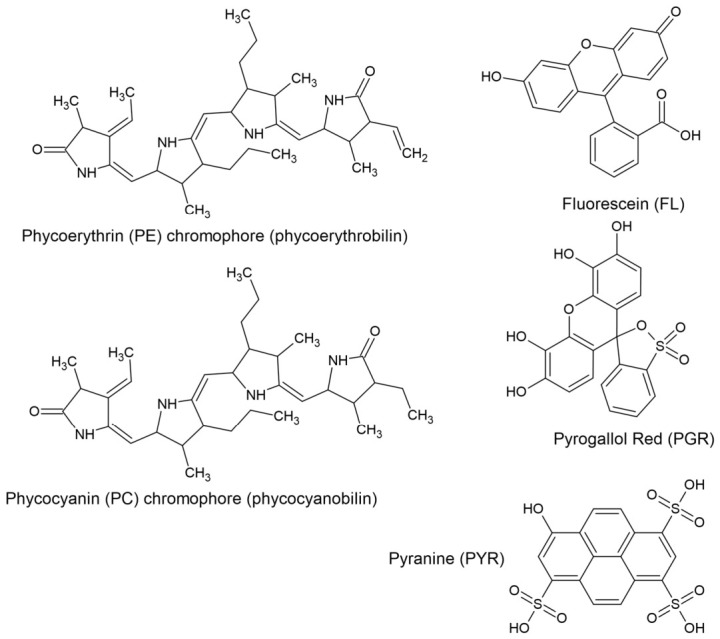
Structures of indicator dyes used most commonly in the ORAC assay.

Although a range of other substrates has been proposed, including 6-carboxyfluoroscein (6-CFL) [39], 2′,7′-dichlorodihydrofluorescein (DCDHFL) [32], Pyrogallol Red (PGR) [40], pyranine (8-hydroxy-1,3,6-pyrene trisulfonic acid) (PYR) [41,42,43], eosins (eosin Y and eosin B) [44], Nile Blue [45], *p*-aminobenzoic acid [46] and BODIPY C11 581/591 (4,4-difluoro-5-(4-phenyl-1,3-butadienyl)-4-bora-3a,4a-diaza-s-indacene-3-undecanoic acid) [32,47], fluorescein (FL) remains the most common indicator in the ORAC assay (Figure 2).

Oxidation of FL by peroxyl radicals is complex. In the first step, a hydrogen of the phenol group is abstracted by a peroxyl radical, forming the fluorescein phenoxyl radical (FL^•^) that undergoes further reactions resulting in the formation of non-fluorescent products, but can also be repaired by antioxidants to restore the original form of the molecule [19].

The reaction of oxidation of the fluorescent probe is driven to completion. In the classic ORAC assay, fluorescence is measured until it decreases to the baseline for all samples. Antioxidants present in the sample protect fluorescein from bleaching. The extent of protection is a measure of the antioxidant activity/capacity of the sample (it has been suggested to use the term “antioxidant activity” for a defined compound and the term “(total) antioxidant capacity” for a sample containing a mixture of unidentified antioxidants [2]).

**Figure 3:**
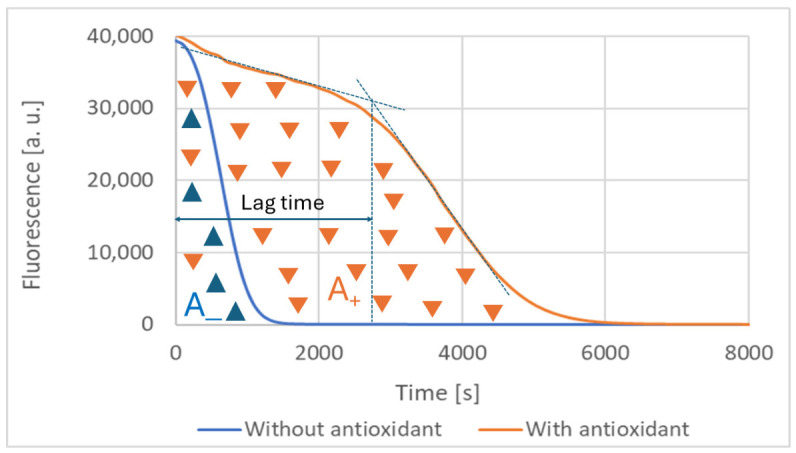
Principle of the ORAC assay. The sum of fluorescence readings of a sample containing an indicator, AAPH and an antioxidant (area under the fluorescence decay curve) (A_+_) minus the sum of fluorescence readings of a sample containing no antioxidant (A_) is a measure of protection of the indicator by the antioxidant, and thus of its antioxidant activity. The lag time, estimated from the intersection of tangents to the initial slope of fluorescence decrease and to the maximal slope of fluorescence decrease, may be another measure of the antioxidant activity. Blue triangles mark A_ while orange triangles mark A_+_.

The parameter used most commonly is the “area under the curve” (AUC), i.e., the sum of fluorescence intensities measured for a given sample during the whole kinetic assay. The net protection areas (AUC for an antioxidant minus AUC for a blank containing no antioxidant) of tested samples are compared to that of the Trolox (a water-soluble vitamin E analog) standard, and results (“ORAC values”) are reported as Trolox equivalents. One ORAC was defined as the net protection area provided by 1 µM (final) Trolox [15]. The ORAC values depend on the probe used for the assay (see Section 6). Alternatively, if a distinct lag period is observed (the time from the beginning of the measurement to the beginning of the phase of the fluorescence decay), its length may be another measure of the antioxidant activity/capacity (Figure 3).

With some indicators, such as DHDCFL or BODIPY C11, the fluorescence increases rather than decreases in the course of the reaction. In such cases, the completion of the reaction is more difficult or even impossible to assess.

A method for determining competitive scavenging of AAPH-generated radicals by comparing the intensities of EPR signals of a spin trap in the absence and presence of antioxidants is referred to as the ORAC-ESR method. In this method, however, the radicals are generated not by thermal decomposition of AAPH but by its UV-photolysis, followed by an instantaneous measurement of the signal intensity [48,49]. One inherent drawback of this assay is that many antioxidants react with both the nitroxide spin adduct and the free radical. Therefore, it is difficult to determine whether the decrease in the formation of spin adducts is due to the scavenging of the free radical or to the reduction of the spin adduct [50].

Disadvantages of the assay have been pointed out by various authors. It was argued that the mere integration of the fluorescein signal (AUC) to represent antioxidant properties has several limitations. The assay does not measure reaction rates but the sum of reactions of antioxidants present in a sample, not differentiating between reaction rate and radical-scavenging efficiency. It is difficult to set a reaction time that compromises between the kinetic complexities of the different antioxidants. In any case, a part of the kinetic information is lost. While assays based on the reduction of stable free radicals or Fe3+ may reflect mainly fast reactions, especially when run for short times, the ORAC assay also includes slower reactions but may underestimate fast reactors and yield positively biased results for slowly reacting antioxidants [51,52,53]. This, however, may not be a drawback of the method if addressing a situation in situ where both fast and low antioxidants may contribute to the antioxidant effect. The share of slow reactions can be expected to increase with extending reaction time (i.e., lowering AAPH concentration).

Undoubtedly, ORAC is a HAT-based assay that has a mechanistic similarity to the peroxidation of biological molecules in situ. However, under the assay conditions, the concentration of the substrate (in this case, a fluorescent probe) is comparable or smaller than the concentration of antioxidants, in contrast to biological systems or food products, where antioxidants are usually (though not always) present in much smaller amounts than substrates protected by them [54].

It has been argued that the units derived from the time-integrated fluorescein signals do not correspond to established chemical or physical quantities and are arbitrary. Moreover, different indicators produce different values of the ORAC units, and the ORAC protocol, even with the same indicator, is subject to variations across different laboratories, leading to inconsistent ORAC values.

## 3. Mechanism of the Assay

A simple picture of the mechanism of the assay is that antioxidants compete with the probe for peroxyl radicals ROO^•^ formed upon AAPH decomposition:

AAPH + O_2_ → 2 ROO^•^ + N_2_ (2)

ROO^•^ + target → ROOH + target^•^ (3)

ROO^•^ + AH → ROOH + A^•^ (4)

where target represents PHE, FL, or another probe and AH is an antioxidant.

Dorta et al. [55] postulated that alkoxyl radicals (RO^•^) rather than peroxyl radicals play a dominant role in the ORAC-FL assay, proposing the following reaction set:

2 ROO^•^ → ROO-OOR (5)

ROO-OOR → 2 RO^•^ + O_2_ (6)

FL-H + RO^•^ → FL^•^ + ROH (7)

AH + RO^•^ → A^•^ + ROH (8)

where FL-H is fluorescein, FL^•^ is the fluoresceinyl free radical, AH is an antioxidant and A^•^ is an antioxidant free radical (the symbol FL-H is used instead of FL to enable illustrating of hydrogen atom abstraction by oxidizing radicals).

Alkoxyl radicals RO^•^ have a higher redox potential than peroxyl radicals ROO^•^ (ca 1.6 vs ca 1.0 V) [56] so they should be expected to be more reactive (although kinetic factors, apart from thermodynamic ones, govern the reactivity of various compounds [57]). If the FL concentration is low (usually about 70 nM), it can be assumed that FL is only removed by alkoxyl radicals. The FL consumption rate corresponds to ca. 10% of the total rate of radicals associated with the AAPH thermolysis. The sequence of ORAC values obtained with FL is Trolox < sinapic acid < coumaric acid, while the sequence of reactivities with peroxyl radicals follows a reverse order [55].

Asma et al. [29] proposed a kinetic model of the ORAC assay, enabling an insight into its mechanism. They considered nine reactions (Table 1).

The model, in contrast to the previous one, does not consider the reactions of alkoxyl radicals with antioxidants. The reaction of fluorescein with ROO^•^ is considered negligible based on reaction with tetrahydrofuran, a compound that rapidly reacts with peroxyl radicals. The measurement of O_2_ consumption during tetrahydrofuran oxidation by peroxyl radicals in the presence of fluorescein showed a negligible affinity of fluorescein for peroxyl radicals [29].

Antioxidants may participate in two steps of the process: by neutralizing peroxyl radicals (Step 5) and by recovering fluorescein from the fluoresceinyl radical (Step 6). Step 5 may involve either direct hydrogen atom transfer or electron transfer followed by protonation/deprotonation of the reactants [58]. Step (6) is rapid and is primarily governed by the equilibrium constant K_6_. Reaction (6) is assumed to be rapid but reversible and characterized by an equilibrium constant K_6_ = k_6_/k_−6_, where k_6_ and k_−6_ represent rate constants for the forward and reverse reactions, respectively [59].

The equilibrium constant K_6_ is related to the difference in the one-electron redox potentials of the fluoresceinyl radical/fluorescein redox pair E(_FL•,H+/FLH_) and of the antioxidant radical/antioxidant redox pair E(_A•,H+/AH_) [59,60]. Lower values of K_6_ indicate a greater electron-donating activity, and, thus, a greater ability to regenerate fluorescein. Trolox showed a high repair capability for fluorescein. This ability is linked to the low redox potential of the Trolox radical/Trolox couple (E_p,a_ = 80 mV) [61], which is significantly lower (more reducing) than that of the fluoresceinyl radical/fluorescein couple (E_p,a_ = 750 mV) [62]. Among the monophenolic cinnamic acids compared, K_6_ values decreased in the following order: sinapic acid > ferulic acid > *p*-coumaric acid. Sinapic acid, with a lower reduction potential (E_p,a_ = 188 mV), exhibited the greatest regenerative potential, exceeding that of ferulic acid (E_p,a_ = 335 mV) and *p*-coumaric acid (E_p,a_ = 737 mV) [63]. Assuming fixed values for some rate constants, the kinetic model finds values of k_5_ and K_6_ providing the best fit to experimental curves of fluorescence decay for several antioxidants [9,29]. Among the phenolic acids studied, the highest value of antioxidant reactivity for peroxyl radicals k_5_ (6.1 × 10^4^ M^−1^ s^−1^) was found for chlorogenic acid, but it was still lower than that for Trolox (4.0 × 10^5^ M^−1^ s^−1^). The order of antioxidant reactivity of phenolic acids and Trolox based on k_5_ values does not correspond to that based on the AUC values (*p*-coumaric acid > ferulic acid > sinapic acid > Trolox) [29].

Alternative models of ORAC reactions will perhaps be proposed.

When the reactivity of an antioxidant with radicals is higher than that of the probe, the lag phase appears [50]. Its length is determined by the amount of radicals scavenged by the antioxidant, i.e., concentration and stoichiometric number of the antioxidant:

lag time = n × [AH] × R_i_ (9)

where n is the stoichiometric number (the number of radicals scavenged by each antioxidant molecule, n = 2 for Trolox) and [AH] is the antioxidant concentration. With FL and PYR, the lag time was proportional to the concentration of antioxidants (Trolox or uric acid) [64].

The rate of radical flux is expressed by

R_i_ = 2 × e × k_d_ × [AAPH] (10)

where e is the efficiency of free radical production [64,65]. It was found that the lag time in the ORAC-FL assay becomes less pronounced with increasing oxidation potential of the antioxidant, and it has been suggested that this is due to the antioxidant repairing fluorescein during this phase [59]. In agreement with this prediction, a correlation was observed between the anodic peak potential and the lag time of five antioxidants [66].

The occurrence of the lag phase in the ORAC-PYR [67,68] has been explained in the same way, by repair reactions of antioxidants (AH) with the pyranine radical (PYR^•^):

PYR + ROO^•^ → PYR^•^ + ROOH (11)

AH + PYR^•^ → PYR + A^•^ (12)

Less reactive antioxidants protect PYR without producing lag times in the kinetics, by a dual mechanism involving both competition for ROO^•^ and PYR^•^ repair [69].

Both AUC and the lag time can be used for the determination of antioxidant activity/capacity. In the ORAC-FL assay, the ORAC values obtained from the lag time assessment were generally lower than those based on AUC values, except for ascorbic acid. The ratio of ORAC values obtained from the lag time to that determined from AUC was 0.98, 0.69, 0.90, 0.91, and 0.70 for ascorbic acid, glutathione, gallic acid, quercetin and caffeic acid [53]. Lag time was also used to estimate antioxidant activity in the ORAC-PYR assay [41,42,43].

PGR at the concentrations used (much higher with respect to fluorescein) has higher reactivity toward peroxyl radicals, and all ROO^•^ generated by the thermolysis of AAPH are neutralized, minimizing the formation of RO^•^ [69]. In ORAC-PGR, only ascorbic acid inhibited the consumption of PGR efficiently enough to induce a lag time. Glutathione, uric acid and human serum albumin did not induce a lag time, while the lag time induced by Trolox was not well resolved [64,70,71]. When ORAC-PGR was applied to herbal and tea infusions [72] and wines [70], no lag time was observed. Lag time was also induced by blood plasma and urine, and disappeared after treatment of plasma or urine with ascorbate oxidase [71]. Ascorbate was responsible for the appearance of the lag time in ascorbate-rich raspberry extract; the lag time was not observed in the ORAC assay of blackberry and blueberry extracts, having lower ascorbate content. Pre-treatment with ascorbate oxidase completely removed the induction time in the raspberry extract. Therefore, lag time in the PGR-ORAC was proposed as a measure of ascorbate content in the extracts. The ascorbate concentration determined from the lag time showed reasonable agreement with the HPLC assay result [73].

The Bors criteria of flavonoid reactivity did not correlate with their activity in the ORAC-FL assay [74]. However, analysis of the dependence of ORAC values of a range of antioxidants on their minimum bond dissociation energy (BDE) value (the lowest among the BDE values of C–H and O–H bonds in a molecule) showed that the regression coefficient was highly significant for both phenols di-substituted at the *ortho* position and phenols monosubstituted at the *ortho* position. These results imply a strong linear relationship between the BDE and hydrophilic ORAC values within each homogeneous cluster of compounds. Neither ionization potential nor proton affinity showed a clear trend like BDE. However, steric factors and solubility should also be taken into account [75].

## 4. ORAC for Lipophilic Antioxidants (L-ORAC)

The aqueous medium of the classic ORAC assay makes it weakly sensitive or insensitive to lipophilic antioxidants. Therefore, adaptations of the assay to measure hydrophobic antioxidants have been proposed.

Prior et al. described a method to assay both hydrophilic and hydrophobic antioxidants based on the addition of ethanol and water to a sample (blood plasma or serum), extraction with hexane, phase separation, and a subsequent extraction of the residue with hexane. In the aqueous phase, protein was precipitated with perchloric acid for the standard (hydrophilic) ORAC assay. The procedure for food samples included the extraction of a lyophilized sample with hexane, evaporation of hexane and extraction of the residue with acetone/water/acetic acid and sonication. The aqueous and hexane fractions were analyzed by hydrophilic ORAC and lipophilic (L-ORAC) assays, respectively. In the lipophilic assay, the dried hexane extract is dissolved in 7% randomly methylated cyclodextrin (RMCD) solution in 50% acetone/50% water, *v*/*v*, and further diluted with the RMCD solution [21].

β-Cyclodextrin (β-CD) is a cyclic hexamer of glucose residues. In RMCDs, some hydroxyl groups are randomly substituted with methoxy groups. RMCDs encapsulate a lipophilic molecule to form a water-soluble inclusion complex. Hydroxypropyl-*β*-cyclodextrin has been mainly used in L-ORAC-FL measurements. However, this approach is not free from drawbacks; it has been reported that cyclodextrins may bind antioxidants, making their reactive groups unavailable for reaction, and bind also fluorescein and AAPH, affecting ORAC measurements [76,77,78,79].

Lipophilic antioxidants were found to represent 33.1–38.2 of the total antioxidant capacity of the protein-free blood plasma [21]. In vegetables, the lipophilic antioxidants as estimated with L-ORAC using fluorescein varied from 3.85% (russet potato) to 18.6% (baby carrot) of the total antioxidant capacity [24]. However, in spices the share of L-ORAC may be much higher: 59.4% in black pepper (whole peppercorn), 74.9% in turmeric, and 75.9% in ground ginger [23].

Other approaches to ORAC assay for lipophilic antioxidants employed hydrophobic azo initiators, AIPH, AMVN, and MeO-AMVN, and BODIPY 581/591 C11, PGR, or PYR as an indicator probes either in a lipophilic medium (octane:butyronitrile [30]) or dimethylsulfoxide: butyronitrile mixture [47]) or in aqueous medium containing dioleoylphosphatidylcholine (DOPC) [30] or methyl palmitate and methyl linoleate [41] liposomal suspensions.

## 5. Technical Considerations

The use of a multiwell plate and a plate reader already means a considerable automation of the assay, but further automation was proposed. The assay has been fully automated for the COBAS FARA II centrifugal analyzer with a fluorescence-measuring attachment (Roche Diagnostic System Inc., Branchburg, NJ, USA) [18,80], resulting in improved efficiency through a robotic eight-channel liquid-handling system and a microplate fluorescence reader.

Apparently, the assay is quite reproducible. An inter-laboratory comparison of two versions of hydrophilic ORAC-FL in 14 laboratories yielded HorRat values of 0.40 to 1.93 [81]. However, results obtained in various laboratories both for single compounds and for similar food products can differ considerably, as discussed in Section 6. What may be the reasons for these discrepancies?

The original ORAC protocols have been modified in various laboratories, so the family of protocols has been used as exemplified in Table 2 for H-ORAC and in Table 3 for L-ORAC. Modifications, apart from the use of new substrates, concerned changes in the concentrations of the probe and of an azo initiator, pH, buffer concentration, and, rarely, the temperature. Interestingly, while the buffer pH varied between 7.0 and 7.4 in the H-ORAC-FL assay, the buffer concentration was mostly kept constant (75 mM). The concentration of AAPH used is also variable. As the decomposition of AAPH is the rate-limiting reaction of ORAC, some authors used higher AAPH concentrations to shorten the duration of the assay.

While compiling these data, surprisingly often we found incomplete or ambiguous descriptions of conditions that suggest appealing to authors, reviewers, and editors to check the completeness of the method description in the manuscripts.

The pH of the ORAC assay, close to the physiological pH, has been listed among the advantages of the method [136], but it also contributes to the sensitivity of the ORAC-FL assay. The fluorescence intensity of FL is pH-sensitive. At pH 7.4 it exists in solution mainly as a dianion, (pK of the carboxyl group is 4.31, and pK of the phenol group is 6.43 [35]). The dianionic form of FL has the highest fluorescence. When pH drops below 7, its intensity decreases greatly; therefore, a phosphate buffer, pH 7.4 is most often used for the assay [19]. Sometimes, the assay is run at pH 7.0 or 7.2 (Table 2). As it can be calculated from the Henderson-Hasselbalch equation, at pH 7.4, 90% of FL is in the dianionic form while at pH 7.0, 79% of FL occurs as the dianion, which means a somewhat lower fluorescence, but this difference is devoid of practical significance.

It has been argued that the pH range of 7.0–7.4 is physiological for animal organisms but not for plant vacuoles, where many antioxidant metabolites are accumulated and are more stable. Some authors [86] used a lower pH (5.5) to prevent degradation of the flavanols and *β*-PE as a fluorescent probe. Others used fluorescein at a similarly low pH (5.44) [87].

The use of PGR is based on the measurement of absorbance, not fluorescence decay, so it requires higher concentrations of the probe [40,41,73,118,134]. An absorptiometric ORAC assay using PYR was also employed [43], requiring, of course, higher PYR concentrations than a fluorometric assay [67,68].

The concentration of fluorescein should be kept as low as possible, not only to shorten the reaction but also due to the concentration-dependent self-quenching of the fluorescence, which was reported to occur at fluorescein concentrations even as low as 1.9 nM [51]. Fluorescence self-quenching introduces some error in the determination of the fluorescence decay. A simple indication of the occurrence of fluorescein self-quenching in a system is the course of the initial part of the fluorescence decay curve: an increase in the time course fluorescence values proves self-quenching at the initial fluorescence concentration. Nevertheless, the indicator concentration must be sufficient to achieve reasonable fluorescence and depends on the available equipment, including filters. Using a filter that transmits fluorescence shifted from the fluorescence peak maximum will decrease the sensitivity of the measurement. Most commonly, 70 nM fluorescein and 10 mM AAPH are used, but the concentration ranges reported by different authors are quite broad (Table 2 and Table 3).

Solvent evaporation may be a problem during a long assay at 37 °C. It does not change the composition of the reaction mixture (except for volatile compounds) but increases their concentration, which can affect the reaction kinetics. Running an assay in a plate with a cover does not provide fully hermetic conditions; moreover, water condensation on the cover may disturb the measurements. Sealing the plate with an RT-PCR sealing film may be a good solution [135].

The assay is temperature-sensitive, and thus, small differences in temperature adversely affect the reproducibility of the method. As the decomposition of AAPH and other azo initiators is temperature-dependent, running the assay at room temperature would be impractical, as it would require too long a time. The assay is almost always run at 37 °C with rare exceptions. One is an attempt to combine ESI-HRMS separation of antioxidants with the ORAC-FL assay, where the temperature of 60 °C was used to accelerate the reaction [137]. A similar modification, using the FIAlab SIChrom equipment (ORAC-SIA method) elevated the reaction temperature to 70 °C, reducing the analysis time to 5 min per sample [138].

Very careful control of ORAC reaction temperature is critical. Consistent generation of radicals requires accurate and reproducible temperature control to ensure timely and complete decomposition of the azo initiator. When required temperatures are not reached, reactions are slow and incomplete, and results are poorly reproducible. Even more problematic, slow reactions can be misinterpreted as increased radical scavenging, leading to an overestimation of antioxidant activity. Obtaining required temperatures with plate readers is difficult. Plastic plates (e.g., 96 wells) are good insulators, so temperatures of most ovens must be set at some higher point to ensure that the appropriate temperature in the wells is reached [51].

Due to the poor thermal conductivity of the polypropylene plate, possible temperature inhomogeneity may occur from well to well, causing considerable variations in the ORAC values. The difference between the maximum and minimum ORAC values within a 96-well plate containing the same concentration of Trolox was found to be 27% of the mean value. When the outer wells were not used, the difference between the highest and the lowest values was below 15% of the mean. Even if external wells were discarded, a gradient in results was observed, with values increasing from the edge to the center of the plate, suggesting that the temperature was probably not identical in all wells. The sealing of the wells by a plastic film allowed a further reduction in the edge effect (around 2.5%) [139]. As a simple way to eliminate the temperature inhomogeneity, pre-heating of the plate at 37 °C for at least 10–15 min before the addition of AAPH has been suggested [18].

A very careful control of oxygen is important. Full and reproducible oxygenation is required for the azide reaction to efficiently generate oxygen-centered radicals. However, the solubility of oxygen declines as temperature increases, so oxygen becomes depleted when solutions are pre-warmed before runs, during thermal equilibration in ovens, and when the reaction is run over long times due to strong antioxidant inhibition. Variations in handling and heating times between samples cause considerable variability in the concentration of dissolved oxygen, causing inconsistent results. With insufficient oxygen, reactions are slow, variable, and do not run to completion [51].

Another problem in the assay that can also be responsible for an underestimation or overestimation of antioxidant (ORAC) activity of about 5–20% if the delay in the AAPH injection between wells in the microplate is not considered. Two solutions have been proposed to eliminate this effect: (i) determination of a correction factor for each well after the calibration of the 96-well plates using a Trolox standard solution, or (ii) a symmetrical distribution of technical replicates of the biological samples to compensate for early and late kinetic reads in the 96-well plates [140].

Autoxidation of polyphenols, ascorbate, and thiols [141,142] can compromise certain antioxidant measurement values. One strategy to reduce the interference of metal ions (which accelerate this autoxidation) in ORAC measurements is the addition of a metal chelator. Inclusion of 80 µM EDTA in the reaction medium increased the ORAC values of antioxidants, from 9.72 to 13.5 for quercetin and from 0.38 to 0.72 for ascorbic acid [116]. However, the reactivity of EDTA with peroxyl radicals, contributing to the apparent antioxidant activity of an antioxidant studied, cannot be excluded.

Phenolic, quinone, and aromatic rings in the FL structure provide multiple sites and modes for nonradical associations that block normal reactions with antioxidants. Polyphenol binding may block reactive OH groups and stabilize FL during reactions. Such interactions between antioxidants and FL mostly increase the apparent radical quenching activity, as reflected by exceptionally long reaction times and implausibly high ORAC values or higher ORAC values with more dilute antioxidants. Alternatively, if the antioxidant and FL fluorescence excitation or emission manifolds overlap, the FL–antioxidant binding can either stabilize or reduce fluorescence emissions. Interaction interferences may be tested by using appropriate blanks with extracts plus FL and analyzing overlaps of FL and phenol excitation and emission manifolds in the fluorescence spectra [51].

The presence of a fluorescent substance in the analyzed sample can be a potential source of interference. However, very few substances emit considerable fluorescence at low micromolar concentrations at the excitation and emission wavelengths employed. In case such a problem appears, comparison of spectra of the studied compound (or its fluorescent product) and the indicator, estimation of the contribution of this reactant to the measured fluorescence and its changes inferred from measurements of its fluorescence at the fluorescence maximum in the course of the reaction, and subtraction of this contribution can be recommended. The same procedure is advisable for the interference of light-absorbing compounds in absorptiometric versions of ORAC.

Inclusion of organic solvents may affect the measured parameters. Lag time and AUC values were higher in the 5% ethanol-aqueous medium with respect to the aqueous buffer, indicating that the reaction between Trolox and AAPH-derived radicals was delayed in organic media [53]. It can be attributed either to solubility enhancement (e.g., quercetin and caffeic acid), to changes in the H-atom donor abilities of compounds [143], or to deprotonation of reactive hydroxyl groups of FL [53].

However, the main reason for the variability of the results of the ORAC assay seems to lie in the extraction of antioxidants from solid samples [132,133,144]. It is tacitly assumed that the antioxidant content in the extract corresponds to its content in the material subject to extraction, which may lead to a significant underestimation of the antioxidant capacity of the original material. For example, the ORAC value of the cyclohexane extract of the mushroom *Inonotus hispidus* was 7.50 mmol TE/kg while that of the methanol extract was 290.0 mmol TE/kg [132]. Depending on the solvent used for the extraction, ORAC of litchi fruit pulp ranged from 10.8 to 34.1 mmol TE/kg [145]. Aqueous extracts of *Stevia* leaves had higher antioxidant activity compared to hydroalcoholic or organic extracts [146]. However, this conclusion may not be valid for other materials, depending on the composition and structure of antioxidants. A QUENCHER method, based on the direct measurement of solid samples introduced into the reaction medium [147,148], is an interesting alternative; however, it still leaves the question of the solubility of antioxidants in the reaction medium and their availability to the AAPH-generated radicals, if matrix-bound. In a *Nature Protocols* article, Gillespie et al. [105] recommended the extraction of plant materials frozen in liquid nitrogen by homogenization with carbide beads with ice-cold 50% acetone. Usually, the highest value obtained in extracts using various solvents is assumed to represent the ORAC value of the examined material, or the same extraction procedure is used when comparing various materials of different compositions of antioxidant compounds. It can still significantly underestimate the real content of antioxidants. This, of course, is the drawback of all methods of estimation of TAC.

What can be frustrating for beginners in this method is the loss of linearity between the antioxidant concentration and AUC with increasing antioxidant concentration. Usually, there is a linear relationship (or a linear range of relationship) between the AUC and antioxidant concentration. Sometimes the ORAC values are calculated by using a quadratic regression equation to describe the relationship between the antioxidant concentration and AUC as a measure of antioxidant activity (capacity)

*AUC* = *a* [(*antioxidant concentration*)^2^] + *b* (*antioxidant concentration*) + *c* (13)

where the *a*, *b* and *c* parameters are determined by optimization of the fitting of the experimental data to Equation (13) [23]. A linear regression was used in the range of 6.25–50 μM Trolox, although the use of a quadratic regression extended slightly the dynamic range of the assay [37]. Carvalho et al. found that the linear ranges of the dependence of both AUC and lag time on the antioxidant concentrations were 2.0–10.0 µM for Trolox and gallic acid, 2.0–8.0 µM for GSH, 3.5–9.0 µM for ascorbic acid, 0.40–2.0 µM for quercetin, and 0.40–1.0 µM for caffeic acid [53].

## 6. Some Miscellaneous Results

Exemplary ORAC values for pure antioxidants collected from the literature, expressed in moles of Trolox equivalents/mole compound, are compiled in Table 4. Please note that the ORAC values were obtained using different assays, but even for the same assay, the huge variability of results reported by various authors is surprising. Apart from the factors discussed in the previous section, the purity of reagents used and problems with the solubility of hydrophobic compounds, not easy to detect when working with their low concentrations, may contribute to this effect. In any case, comparison of data for various compounds from the same laboratory should provide the most reliable estimate of their relative antioxidant potency.

Unfortunately, there is no universal coefficient that allows the recalculation of results obtained with one assay to those of another. Ou et al., comparing reactivities of nine compounds in the ORAC-PE and ORAC-FL found that the ORAC values in ORAC-FL of the same compounds were 1.65–3.52 times higher in the ORAC-FL assay, depending on the compound [19].

When comparing ORAC-FL with ORAC-PGR, the values of PGR-ORAC for herbal infusions were nearly 80 times lower than those obtained with ORAC-FL. Analogous data for individual antioxidants were very divergent [72]. Analysis of data collected in Table 3, and using mean values obtained with both assays by various authors revealed a remarkable dispersion of ORAC-FL/ORAC-PGR values: 0.07 (ascorbic acid), 0.09 (GSH), 0.46 (ECG), 0.48 (EGCG), 0.79 (quercetin), 0.83 (kaempferol), 1.65 (isorhamnetin), 8.58 (genistein), 9.40 (apigenin), 10.21 (daidzein), 11.59 (catechin), 12.58 (naringenin), 14.81 (epicatechin), 17.01 (caffeic acid), 17.40 (hesperitin), 32.73 (uric acid), 43.48 (rutin), and even 123.3 (protocatechuic acid).

The data obtained for various food products and some medicinal plants are compiled in Table 5. Again, the discrepancies for apparently the same products are sometimes striking and point to the necessity of reporting details (e.g., varieties of plants, conditions of growth and storage), although the well-known dependence of the antioxidant content of plant-derived foods on such factors as fertilization, climate, weather, time of collection and storage conditions will always affect the results. However, as mentioned earlier, the extraction protocol seems to be the main culprit for the differences in the results of studies of the same materials. Inspection of Table 5 suggests a cautious approach to the published data on the TAC of food products.

Some miscellaneous results can be mentioned. ORAC values of apples decreased during their growth and ripening [246]. In contrast, ORAC values of seriguela (*Spondias purpurea*) fruit pulp increased during fruit maturation. ORAC values increased during cabbage fermentation and were higher for sauerkraut than for pickled cabbage [235]. ORAC values decreased during the ripening of jujube (*Ziziphus mauritiana*) fruit [247].

Cooking generally reduces ORAC, but there are cases where the ORAC value increases upon cooking (Table 5). Cooking and frying of chicken eggs decreased the yolk ORAC values; a decrease was also found during storage for up to 6 weeks [206]. Simulated digestion of sesame seeds led to an increase in the ORAC values, the highest after the simulated small intestine digestion [248]. The ORAC value of sweet whey was considerably increased after simulated digestion [243]. Comparison of the ORAC of conventional Arabica coffee and coffee recovered from the dung of civets and elephants did not show striking differences [203]. Irradiation of walnuts with a high dose of ϒ-radiation (25 kGy) considerably increased their ORAC value [242]. Donor human milk stored for 5 months under hyperbaric conditions (pressures of 60−130 MPa) at subzero temperatures (−5 to −12 °C) retained higher ORAC values compared with pasteurized milk stored at −20 °C, demonstrating the superiority of the hyperbaric treatment [249].

In some cases, results of the ORAC assay may be applied as predictive factors. For example, the proneness of white wines to atypical aging was found to be negatively correlated with their ORAC values [250]. Wines with higher ORAC values were less prone to developing atypical accelerated aging [251]. The ORAC value of methanol/water extracts of oils was proposed as a quality index for virgin olive oil because it measures the effectiveness of phenolic compounds in protecting against peroxyl radicals [252].

## 7. Importance, Use, and Abuse of ORAC Values of Food Products

ORAC-FL data on about 300 foods were assembled into a database and made available on the U.S. Department of Agriculture (USDA) database in 2007, based upon published research, primarily provided by Wu et al. [23,24] and updated in 2010 [164]. The growing interest of consumers in antioxidants induced public interest in these data and caused many consumers to compose a diet on the basis of the antioxidant capacity of foods. Some nutraceutical manufacturers included ORAC values on product labels [38]. Supplement products have been competing against each other for the title of “Highest ORAC Value” [253].

However, the competitive use of ORAC values has brought misconceptions and misuse. At one point, the USDA recommended that 3000–5000 ORAC units be consumed per day for health. 5000 ORAC units can be obtained from one Granny Smith apple or one ounce of pecans, but few people would consider that either of these food quantities contains sufficient antioxidants. However, since the reaction of tocopherol (vitamin E) with fluorescein is comparable to that of Trolox, it has been argued that 5000 ORAC units is equivalent to about 3230 IU of tocopherol daily. Compared to the 30 IU needed to prevent deficiency and the 400 IU maximum recommended supplement level, 5000 ORAC units of tocopherol, totally absorbed, could be a potentially dangerous overdose [51]. This argument seems exaggerated since consumers would use ORAC values for food products containing a complex set of antioxidants rather than for a single vitamin supplement.

Nevertheless, in 2010, the database was withdrawn by the USDA. Reasons given for its withdrawal were: (1) There is “mounting evidence that the values indicating antioxidant capacity have no relevance to the effects of specific bioactive compounds, including polyphenols, on human health”; and (2) “There is no evidence that the beneficial effects of polyphenol-rich foods can be attributed to the antioxidant properties of these foods. The data for antioxidant capacity of foods generated by in vitro (test-tube) methods cannot be extrapolated to in vivo (human) effects, and the clinical trials to test the benefits of dietary antioxidants have produced mixed results. We know now that antioxidant molecules in food have a wide range of functions, many of which are unrelated to the ability to absorb free radicals” [38,253].

Still, there is significant evidence that the ORAC value of a diet is associated with beneficial health effects. High dietary ORAC negatively correlated with the risk of endometrial cancer [254], breast cancer (especially among premenopausal women) [255], hypertension [256], and preeclampsia. Women in the highest tertile of dietary ORAC were 67% less likely to have preeclampsia than those in the lowest tertile of ORAC [257]. In patients with liver cirrhosis, dietary TAC estimated by ORAC had a significant inverse association with disease severity [258]. The diet of Italian children with food allergies had a lower antioxidant potential (expressed as ORAC value) than the diet of healthy children, regardless of the allergenic food excluded from the diet, apparently due to a reduced variety of the diet [259]. An inverse correlation between dietary ORAC and body mass index (BMI) and waist-to-hip ratio (WHR), and a significant positive correlation between dietary ORAC and plasma HDL were observed in a study of normal, overweight, and obese subjects, apparently associated with the consumption of more fruits and vegetables [260]. Higher dietary ORAC was associated with greater gut microbiota diversity and increased abundance of such taxa as Barnesiella, Coprococcus, Ruminococcus, Parabacteroides, Lachnospiraceae NK4A136 group, and Clostridia UCG-014 group [261]. Even in these cases, ORAC values are an indirect index of food quality; this simple parameter seems quite useful in screening studies.

## 8. Hydroxyl Radical Absorbance Capacity (HORAC), Peroxynitrite Absorbance Capacity (NORAC), Superoxide Anion Absorbance Capacity (SORAC), and Singlet Oxygen Absorbance Capacity (SOAC)

Apart from ORAC measuring the reactivity of antioxidants for peroxyl/alkoxyl radicals, related assays estimating their reactivity for ROS/RNS most common in biological systems and food products (hydroxyl radical, peroxynitrite, superoxide radical and singlet oxygen: HORAC, NORAC, SORAC, and SOAC, respectively, have been proposed.

Ou et al. [20] proposed a method of measurement of “Hydroxyl Radical Prevention (or Absorbance) Capacity” (HORAC). In this procedure, hydroxyl radicals are generated in a Fenton-like reaction between Co^2+^ and hydrogen peroxide:

Co^2+^ + H_2_O_2_ → Co^3+^ + HO^•^ + HO^−^ (14)

and react with fluorescein, causing bleaching of the indicator. Using this method, HORAC values can be determined for various compounds and complex materials, by analogy to ORAC values. There was a criticism, however, of the purposefulness of the estimation of the reactivity of biological or food material with ^•^OH. This radical is so reactive that it will react with the first molecule encountered, and differences in the reactivity of various compounds with this radical have little effect (if any) on the effects of its reaction; proximity of molecules to the site of OH formation, and reactivities of secondary radicals formed are more important [262].

Peroxynitrite (ONOO−) Scavenging (or Absorbance) Capacity (NORAC) measures the peroxynitrite scavenging capacity of biological fluids and food materials, using dihydrorhodamine 123 as a probe and peroxynitrite or 3-morpholino-sydnonimine (SIN-1) as a precursor of peroxynitrite [263]. Superoxide Radical Antioxidant (or Absorbance) Capacity (SORAC) is an assay method used to measure an antioxidant’s ability to scavenge superoxide anions, with dihydroethidium (DHE) as a probe and the xanthine/xanthine oxidase as a superoxide-generating system [263,264]. Singlet Oxygen Scavenging (or Absorbance) Capacity (SOAC) is a method to quantify the ability of antioxidants and food extracts to quench singlet oxygen, with DHE as a probe and Na_2_MoO_4_/H_2_O_2_ as a singlet oxygen source [265]. This set of five assays measuring TAC with respect to various biologically and technologically important oxidants has been termed “ORAC 5.0” [263,265] or multi-radical antioxidant capacity “ORAC_MR5_” [162]. Conditions of these assays are shown in Table 6. However, by no means is the total antioxidant activity of a sample the sum of the five activities measured by 5.0, as the same antioxidants contribute to the results of all assays, albeit with different shares.

## 9. Conclusions

ORAC is a robust assay enabling evaluation of antioxidant activity/capacity under conditions close to physiological, using an oxidant acting in living organisms and during food storage and processing. Several versions of the assay have been proposed, but the one using fluorescein is more often used and should be recommended, as results obtained with various probes are not interconvertible. The use of both hydrophilic and lipophilic ORAC is strongly suggested. Among the various technical factors responsible for the scatter in the results of the antioxidant capacity of food products, the way of extracting antioxidants from the material studied seems the most important; the procedure of Gillespie et al. [105] for plant material could be used as a standard. The standardization of the assay conditions is recommended; we propose final concentrations of fluorescein and AAPH of 40 nM and 12 mM, respectively, unless the sensitivity of the equipment requires the use of a higher fluorescein concentration, and 75 mM phosphate buffer, pH 7.4.

## Figures and Tables

**Table 1:** Reactions considered in the ORAC assay by Asma et al. [29]. From [29], modified.

Step	Reaction	Kinetic Parameter
Initiation (1)	AAPH + O_2_ → 2 ROO^•^	R_i_ = 1.36 × 10^−6^ × [AAPH] M s^−1^
Peroxyl termination (2)	2 ROO^•^ → NRP	k_2_ = 1 × 10^−6^ M^−1^ s^−1^
Alkoxyl formation (3)	2 ROO^•^ → 2 RO^•^ + O_2_	k_3_ = 5 × 10^4^ M^−1^ s^−1^
Fluorescein bleaching (4)	RO^•^ + FL-H → ROH + FL^•^	k_4_ = 1 × 10^7^ M^−1^ s^−1^
Radical scavenging (5)	AH + ROO^•^ → ROOH + A^•^	k_5_ = (1–40) × 10^4^ M^−1^ s^−1^ (variable)
Fluorescein repair (6)	AH + FL^•^ → A^•^ + FL-H	K_6_ = k_6_/k_−6_ = 1–20 (variable)
Alkoxyl termination (7)	2 RO^•^ → NRP	k_7_ = 1 × 10^9^ M^−1^ s^−1^
Antioxidant termination (8)	2 A^•^ → NRP	k_8_ = 1 × 10^8^ M^−1^ s^−1^
Fluoresceinyl radical termination (9)	2 FL^•^ → NRP	k_9_ = 1 × 10^8^ M^−1^ s^−1^

FL-H, fluorescein, NRP, non-radical product.

**Table 2:** Conditions of the hydrophilic ORAC (H-ORAC) assay.

Conditions	Reference
*Probe: β-phycoerythrin (β-PE)*	
16.7 nM *β*-PE, 3 mM AAPH, 75 mM phosphate buffer, pH 7.0, 37 °C, 540/565 nm	[15]
16.7 nM *β*-PE, 8.11 mM AAPH, phosphate buffer, pH 7.0, 37 °C, 485/535 nm	[82]
32 nM *β*-PE, 0.4 mM AAPH, 75 mM phosphate buffer, pH 7.0, 37 °C, 485/528 nm	[83]
32 nM *β*-PE, 4 mM AAPH, 75 mM Na-K phosphate buffer, pH 7.0, 37 °C, 540/565 nm	[84]
2.94 mg/L *β*-PE, 32 mM AAPH, 75 mM phosphate buffer, pH 7.0, 37 °C, 535/560 nm	[85]
3.39 mg/L *β*-PE, 8 mM AAPH, 5 mM sodium acetate buffer, pH 5.5, 37 °C, 535/595 nm	[86]
*Probe: β-phycocyanin (β-PE)*	
16.7 nM *β*-PC, 3 mM AAPH, 75 mM phosphate buffer, pH 7.0, 37 °C, 598/615 nm	[34]
*Probe: fluorescein (FL)*	
0.107 nM FL, 27.3 mM AAPH, 37 °C, 485/520 nm	[87]
0.47 nM FL, 2.72 mM AAPH, 75 mM phosphate buffer, pH 7.4, 485/523 nm	[88]
2.67 nM FL, 17 mM AAPH, 75 mM Na/K phosphate buffer, pH 7.4, 37 °C, 485/530 nm	[89]
3 nM FL, 19.1 mM AAPH	[90]
3.75 nM FL, 14.5 mM AAPH, 75 mM buffer, pH 7.4, 37 °C, 485/535 nm	[91]
6 nM FL, 9.4 mM AAPH, 75 mM phosphate buffer, pH 7.4, 37 °C, 485/528 nm	[92]
6 nM FL, 18.8 mM AAPH, 75 mM phosphate buffer, pH 7.4, 37 °C, 485/528 nm	[93]
7.5 nM FL, 19.1 mM AAPH, 75 mM phosphate buffer, pH 7.4, 37 °C, 485/527 nm	[94]
23.4 nM FL, 20 µL, 24 mM AAPH, 75 mmol/L phosphate buffer, pH 7.4, 37 °C, 485/520 nm	[95]
3 nM FL, 19.1 mM AAPH	[90]
3.75 nM FL, 14.5 mM AAPH, 75 mM buffer, pH 7.4, 37 °C, 485/535 nm	[91]
30 nM FL, 19 mM AAPH, 75 mM phosphate buffer, pH 7.0, 37 °C, 485/520 nm	[96]
31.2 nM FL, 44.2 mM AAPH, 75 mM phosphate buffer, pH 7.0	[97]
35 nM FL, 12 mM AAPH, 75 mM phosphate buffer, pH 7.4, 37 °C, 485/520 nm	[53]
40 nM FL, 17 mM AAPH, 37.5 mM phosphate buffer, pH 7.4, 37 °C, 480/520 nm	[59]
41.3 nM FL, 0.42 mM AAPH, 75 mM sodium phosphate buffer, pH 7.0, 37 °C, 485/520 nm	[98]
42 nM FL, 3.6 mM AAPH, 75 mM phosphate buffer, pH 7.4, 37 °C, 485/520 nm	[99]
45.8 nM FL, 12.8 mM AAPH, 75 mM phosphate buffer, pH 7.4, 37 °C, 485/520 nm	[100,101]
50 nM FL, 12.8 mM AAPH, 75 mM phosphate buffer, pH 7.0, 37 °C, 485/520 nm	[102]
59.1 nM FL, 47.5 mM AAPH, 75 mM phosphate buffer, pH 7.4, 37 °C, 485/520 nm	[103]
60 nM FL, 12 mM AAPH, 37 °C, 485/527 nm	[104]
60 nM FL, 18.8 mM AAPH, 75 mM phosphate buffer, pH 7.0, 37 °C, 485/530 nm	[105]
60 nM FL, 18.8 mM AAPH, 75 mM K-phosphate buffer, pH 7.4, 37 °C, 485/520 nm	[106]
60 nM FL, 50 mM AAPH, 75 mM phosphate buffer, pH 7.4, 37 °C, 485/527 nm	[107]
61.2 nM FL, 19.1 mM AAPH, 75 mM phosphate buffer, pH 7.4, 37 °C, 485/520 nm	[108]
61.2 nM FL, 19.1 mM AAPH, 75 mM phosphate buffer, pH 7.0, 37 °C, 485/525 nm	[109]
61.2 nM FL, 19.1 mM AAPH, 75 mM phosphate buffer, pH 7.4, 485/530 nm	[18]
61.2 nM FL, 25 mM AAPH, 37 °C, 75 mM phosphate buffer, pH 7.0, 485/528 nm	[110]
63 nM FL, 8 mM AAPH, phosphate buffer, pH 7.2, 37 °C, 490/515 nm	[111]
63.7 nM FL, 7.93 mM AAPH, 75 mM phosphate buffer, pH 7.4, 37 °C, 485/528 nm	[81]
70 nM FL, 3.33 mM AAPH, 75 mM phosphate buffer, pH 7.0, 37 °C, 485/520 nm	[112]
70 nM FL, 10 mM AAPH, 75 mM phosphate buffer, pH 7.4, 37 °C, 493 nm/515 nm	[73]
70 nM FL, 12 mM AAPH, 37 °C, 485/520 nm	[113]
71.9 nM FL, 20.3 mM AAPH, 75 mM phosphate buffer, pH 7.4, 37 °C	[114]
73.8 nM FL, 50.8 mM AAPH, 75 mM sodium phosphate buffer, pH 7.0, 485/520 nm	[115]
78 nM FL, 12 mM AAPH, 75 mM phosphate buffer, pH 7.4, 37 °C, 485/520 nm	[116]
79.2 nM FL, 6.63 mM AAPH, phosphate buffer, pH 7.4, 37 °C, 485/528 nm	[117]
80 nM FL, 10 mM AAPH in 75 mM phosphate buffer, pH 7.4, 37 °C, 460/510 nm	[118]
83.4 nM FL, 19.1 mM AAPH, 75 mM phosphate buffer, pH 7.2, 37 °C, 485/535 nm	[119]
152.5 nM FL, 41.8 mM AAPH, 75 mM phosphate buffer, pH 7.4, 37 °C, 485/528 nm	[120]
400 nM FL, 56.3 mM AAPH, 75 mM phosphate buffer, pH 7.4, 37 °C, 485/530 nm	[121]
445.8 nM FL, 12.8 mM AAPH, 75 mM phosphate buffer, pH 7.0, 37 °C, 485/520 nm	[122]
500 nM FL, 73 mM AAPH, 100 mM phosphate buffer, pH 7.4, 37 °C, 450/515 nm	[123]
750 nM FL, 31.3 mM AAPH, 10 mM phosphate, pH 7.4, 37 °C, 485/520 nm	[124]
750 nM FL, 31.3 mM AAPH, 75 mM phosphate buffer, pH 7.4, 37 °C, 485/520 nm	[125]
800 nM FL, 9.9 mM AAPH, 75 mM K-phosphate buffer, pH 7.4, 37 °C, 485/538 nm	[126]
3 µM FL, 21.6 mM AAPH, 37 °C, 75 mM phosphate buffer, pH 7.4, 485/520 nm	[127,128]
3.14 μM FL, 153 mM AAPH, 0.5 mL, 75 mM phosphate buffer, pH 7.2, 37 °C, 493/515 nm	[129]
3.70 µM FL, 8.4 mM AAPH, 75 mM sodium phosphate buffer, pH 7.0, 540/565 nm	[130]
6.38 µM FL, 17.7 mM AAPH, 75 mM phosphate buffer, pH 7.4, 37 °C, 485/535 nm	[131]
10.5 µM FL, 30 mM AAPH, 75 mM phosphate buffer, pH 7.4, 37 °C, 485/520 nm	[132]
11.2 µM FL, 49.2 mM AAPH, Na-phosphate buffer, 32 °C, 540/565 nm	[133]
14.0 µM FL, 4.8 mM AAPH, 75 mM phosphate buffer, pH 7.0, 37 °C	[21]
*Probe: 6-carboxyfluorescein (6-CFL)*	
15 nM 6-CFL, 3 mM AAPH, 75 mM phosphate buffer, pH 7.0, 37 °C, 495/520 nm	[39]
*Probe: 2′,7′-dichlorodihydrofluorescein (DCDHFL)*	
1 µM DCDHFL, 200 µM AAPH, PBS, 485/535 nm	[32]
*Probe: Pyrogallol Red (PGR)*	
5 µM PGR, 10 mM AAPH, 75 mM phosphate buffer, pH 7.4, 37 °C, 540 nm	[40,73,134]
20 µM PGR, 20 mM AAPH, 75 mM phosphate buffer, pH 7.4, 37 °C, 540 nm	[118]
*Probe: pyranine (PYR)*	
5 µM PYR, 10 mM AAPH, 10 mM phosphate buffer, pH 7.0, 37 °C, 460/510 nm	[67,68]
50 µM PYR, 50 mM AAPH, PBS, pH 7.4, 37 °C, 454 nm	[43]

PBS, phosphate-buffered saline; usually, the samples are pre-incubated at 37 °C for at least 15 min before the addition of AAPH.

**Table 3:** Conditions of the lipophilic ORAC (L-ORAC) assay.

Conditions	Reference
3 nM FL, 19.1 mM AAPH, sample diluted in 7% RMCD in acetone/water (50%/50%, *v*/*v*)	[90]
63.7 nM FL, 15.9 mM AAPH, samples diluted with 7% RMCD in 50% acetone, containing 10% DMSO; plate sealed with an RT-PCR sealing film	[135]
14 µM FL, 9.6 mM AAPH solution, sample in 7% RMCD, 50% acetone/50% water *v*/*v* (final) 75 mM phosphate buffer, pH 7.0, 37 °C	[21]
30 µM PGR, 30 mM AIPH, 0.5 M SDS containing methyl palmitate and methyl linoleate (0.5 vol % each) in PBS, pH 7.4, 37 °C, 540 nm	[41]
50 µM PYR, 30 mM AIPH, 0.5 M SDS containing methyl palmitate and methyl linoleate (0.5 vol % each), PBS, pH 7.4, 37 °C, 454 nm	[41]
1.3 nM BODIPY 581/591 C11, 0.26 M AMVN in octane:butyronitrile (9:1, *v*/*v*), 41 °C, 570/600 nm	[30]
80 nM BODIPY 581/591 C11, 4 mM AMVN, 1.8 mM DOPC liposomes, 20 mM Tris–HCl buffer, pH 7.4, 42 °C, 540/600 nm	[30]
2 µM BODIPY C11 581/591, 2 mM MeO-AMVN, PBS, 37 °C, 485/535 nm	[32]

AIPH, 2,2′-azobis[2-(2-imidazolin-2-yl)propane]; AMVN, 2,2′-azobis (2,4-dimethylvaleronitrile); Meo-AMVN, 2,2′-azobis(4-methoxy-2,4-dimethylvaleronitrile).

**Table 4:** Antioxidant activities of various compounds.

Compound	Antioxidant Activity [mol TE/mol]
N-Acetylcysteine	0.40_ORAC-EPR_ [48]
Apigenin	8.2_ORAC-FL_ [55], 8.95_ORAC-FL_ [149], 0.96_ORAC-PGR_ [118], 0.97_ORAC-PGR_ [149]
Ascorbic acid	0.43_ORAC-PE_ [19], 0.52_ORAC-PE_ [15], 0.30_ORAC-FL_ [150], 0.38_ORAC-FL_ [116], 0.50_ORAC-FL_ [128], 0.53_ORAC-FL_ [53], 0.65_ORAC-FL_ [151], 0.95_ORAC-FL_ [19,20], 0.98_ORAC-FL_ [123], 1.11_ORAC-FL_ [152], 9.7_ORAC-PGR_ [71], 10.3_ORAC-PGR_ [153], 10_ORAC-EPR_ [48]
Butylated hydroxytoluene (2,6-*di*-tert-butyl-*p*-cresol, BHT)	0.20_ORAC-FL_ [152], 5.53_L_-_ORAC-FL_ [135]
*t*-Butylhydroxyanisol (BHA)	2.43_ORAC-FL_ [154]
Caffeic acid	1.40_ORAC-PE_ [19], 1.9_ORAC-PC_ [155], 2.64_ORAC-FL_ [150], 3.9_ORAC-FL_ [156], 4.37_ORAC-FL_ [20], 7.19_ORAC-FL_ [81], 8.26_ORAC-FL_ [53], 0.2_ORAC-PGR_ [40], 0.42_ORAC-PGR_ [153], 1.62_ORAC-EPR_ [48]
5-Caffeoylquinic acid	3.5_ORAC-FL_ [156]
Capsaicin	5.03_ORAC-FL_ [152]
*α*-Carotene	0.51_ORAC-BODIPY_ [30]
*β*-Carotene	0.56_ORAC-FL_ [148], 0.27_ORAC-BODIPY_ [30]
Carvacrol	1.35_ORAC-FL_ [130]
Carvone	0.12_ORAC-FL_ [157]
Catechin	2.57_ORAC-PE_ [19], 1.5_ORAC-PC_ [155], 6.40_ORAC-FL_, [20], 6.76_ORAC-FL_ [19], 7.9_ORAC-FL_ [128], 10.41_ORAC-FL_ [81], 10.6_ORAC-FL_ [149], 10.8_ORAC-FL_ [158], 0.20_ORAC-PGR_ [149], 1.32_ORAC-PGR_ [40], 0.71_ORAC-EPR_ [48]
Catechol	3.4_ORAC-FL_ [156]
Chlorogenic acid	1.90_ORAC-PE_ [19], 3.14_ORAC-FL_ [20], 5.3_ORAC-FL_ [128]
Cinnamic acid	0.15_ORAC-FL_ [158]
*p*-Coumaric acid	4.1_ORAC-FL_ [55], 5.00_ORAC-FL_ [158]
Curcumin	1.14_ORAC-FL_ [152]
Cyanidin	4.4_ORAC-FL_ [128]
Cyanidin-3-*O*-galactoside	5.8_ORAC-FL_ [128]
Cyanidin-3-*O*-glucoside	7.3_ORAC-FL_ [128]
Cyanidin-3-*O*-rutinoside	5.5_ORAC-FL_ [128]
Cysteine	0.40_ORAC-FL_ [152]
Daidzein	7.66_ORAC-FL_ [149], 0.75_ORAC-PGR_ [149]
Delphinidin	3.8_ORAC-FL_ [128]
Delphinidin-3-*O*-glucoside	5.9_ORAC-FL_ [128]
2,3-Dihydroxybenzoic acid	4.36_ORAC-FL_, 4.45_ORAC-FL_ [17]
2,4-Dihydroxybenzoic acid	1.72_ORAC-FL_, 2.11_ORAC-FL_ [17]
Epicatechin gallate (ECG)	1.89_ORAC-FL_, 2.34_ORAC-FL_ [17], 3.6_ORAC-FL_ [20], 6.75_ORAC-FL_ [149], 7.84_ORAC-PGR_ [149]
Edavarone	5.65_ORAC-FL_ [152]
Epigallocatechin (EGC)	2.5_ORAC-FL_ [20], 3.1_ORAC-FL_ [128]
Epigallocatechin gallate (EGCG)	3.4_ORAC-FL_ [128], 3.51_ORAC-FL_, 3.66_ORAC-FL_ [17], 4.94_ORAC-FL_ [20], 5.71_ORAC-FL_ [149], 8.79_ORAC-PGR_ [149]
Ellagic acid	2.9_ORAC-FL_ [128], 3.1_ORAC-FL_ [55],
Epicatechin	5.1_ORAC-FL_ [128], 9.37_ORAC-FL_ [158], 9.97_ORAC-FL_ [149], 0.55_ORAC-PGR_ [149],
Ethyl caffeate	0.42_ORAC-PGR_ [153]
Gallocatechin	8.3_ORAC-FL_ [128]
Genistein	2.30_ORAC-PE_ [19], 5.93_ORAC-FL_ [19], 7.32_ORAC-FL_ [149], 0.772_ORAC-PGR_ [149], 0.43_ORAC-EPR_ [48]
Glutathione	0.32_ORAC-PE_ [19], 0.48_ORAC-FL_ [157], 0.49_ORAC-FL_ [53], 0.50_ORAC-FL_ [128], 0.62_ORAC-FL_ [19], 5.62_ORAC-PGR_ [71], 0.57_ORAC-EPR_ [48]
Hesperetin	4.56_ORAC-FL_ [81], 7.97_ORAC-FL_ [149], 0.36_ORAC-PGR_ [149]
Hesperidin	4.5_ORAC-FL_ [128]
*p*-Hydroxybenzoic acid	2.01_ORAC-FL_, 2.38_ORAC-FL_ [17], 6.05_ORAC-FL_ [158]
Hydrocortisone	0.0054_ORAC-FL_ [152]
Hydroxycinnamic acid	2.05_ORAC-FL_, 2.16_ORAC-FL_ [17]
Isoeugenol	2.09_ORAC-FL_ [150]
Isoquercitrin	4.50_ORAC-FL_ [20]
Isorhamnetin	8.07_ORAC-FL_ [149], 4.88_ORAC-PGR_ [149]
Kaempferol	2.57_ORAC-PE_ [154], 2.29_ORAC-FL_, 2.75 _ORAC-FL_ [17], 5.22_ORAC-FL_ [20,155], 6.2_ORAC-FL_ [128], 7.87_ORAC-FL_ [149], 10.2_ORAC-FL_ [154], 4.99_ORAC-PGR_ [149], 8.8_ORAC-PGR_ [154]
Kaempferol-3-*O*-glucoside	6.6_ORAC-FL_ [128]
R-(+)-Limonene	0.05_ORAC-FL_ [130]
Linalool	0.28_ORAC-FL_ [130]
Lutein	0.42_ORAC-BODIPY_ [30]
Methyl gallate	12.0_ORAC-PGR_ [153]
Morin	3.19_ORAC-FL_ [123]
Myricetin	1.8_ORAC-FL_ [55], 3.6_ORAC-FL_ [128], 4.26_ORAC-FL_, 4.64_ORAC-FL_ [17], 5.08_ORAC-FL_ [152]
Myricetin-3-rhamnoside	6.0_ORAC-FL_ [128]
Naringenin	5.6_ORAC-FL_ [128], 8.04_ORAC-FL_ [149], 0.542_ORAC-PGR_ [149]
Naringin	4.11_ORAC-FL_ [123]
Octyl gallate	9.82_ORAC-PGR_ [153]
*ϒ*-Oryzanol	12.4_L_-_ORAC-FL_ [135]
Peptide YLVN	1.12_ORAC-FL_ [159]
Peptide EEHLCFR	0.92_ORAC-FL_ [159]
Peptide TFY	0.37_ORAC-FL_ [159]
Perillyl alcohol	0.68_ORAC-FL_ [157]
*α*-Pinene	0.04_ORAC-FL_ [130]
Probucol	0.29–0.32_ORAC-BODIPY_ [30]
Propyl gallate	12.3_ORAC-PGR_ [153]
Protocatechuic acid	5.14_ORAC-FL_, 5.21_ORAC-FL_ [20], 6.7_ORAC-FL_, 7.61_ORAC-FL_ [158], 0.05_ORAC-PGR_ [40]
Quercetin	2.07_ORAC-PE_ [19], 4.2_ORAC-FL_ [128], 4.38_ORAC-FL_ [20], 5.45_ORAC-FL_ [123], 5.48_ORAC-FL_ [151], 6.46_ORAC-FL_ [17], 7.06_ORAC-FL_, 7.28_ORAC-FL_ [19], 9.51_ORAC-FL_ [149], 9.72_ORAC-FL_ [116] 10.7_ORAC-FL_, 10.9_ORAC-FL_ [152], 16.6_ORAC-FL_ [53], 4.2–4.6_ORAC-DCDHFL_ [39], 8.86_ORAC-PGR_ [149], 8.92_ORAC-PGR_ [118], 11.5_ORAC-PGR_ [40], 11.9_ORAC-PGR_ [153], 7.1_ORAC-EPR_ [48]
Quercetrin	2.70_ORAC-PE_ [19], 3.5_ORAC-PC_ [155], 6.47_ORAC-FL_ [19]
Resveratrol	12_L_-_ORAC-FL_ [160]
Resolvin D1	1.22_ORAC-FL_ [151]
Resolvin D1 methyl ester	3.49_ORAC-FL_ [151]
Resolvin D2	1.38_ORAC-FL_ [151]
Resolvin D2 methyl ester	1.45_ORAC-FL_ [151]
Rutin	1.95_ORAC-PE_ [19], 2.3_ORAC-PC_ [155], 3.80_ORAC-FL_ [123], 4.28_ORAC-FL_ [20], 4.6_ORAC-FL_ [128], 6.01_ORAC-FL_ [19], 7.40_ORAC-FL_ [158], 3.5–4.3_ORAC-DCDHFL_, 0.12_ORAC-PGR_ [40], 1.4_ORAC-EPR_ [48]
Sinapic acid	1.65_ORAC-FL_ [150], 2.66_ORAC-FL_ [158], 2.8_ORAC-FL_ [55]
Syringic acid	1.62_ORAC-FL_ [158]
*α*-Terpineol	2.72_ORAC-FL_ [157]
Thymol	1.37_ORAC-FL_ [130]
α-Tocopherol	0.72–0.76_ORAC-BODIPY_ [30]
*ϒ*-Tocopherol	6.28_L_-_ORAC-FL_ [135]
Tryptophan	2.5_ORAC-FL_ [161]
Tyrosine	0.004_ORAC-PC_ [155]
Uric acid	0.92_ORAC-PE_ [15], 0.72_ORAC-FL_ [150], 0.93–1.07_ORAC-DCDHFL_ [39], 0.022_ORAC-PGR_ [71], 2.2_ORAC-EPR_ [48]
2″-*O*-β-D-xylosylvitexin	5.75_ORAC-FL_, 0.552_ORAC-PGR_ [118]
Vanillic acid	3.70_ORAC-FL_ [158]

DCDHFL, 2′,7′-dichlorodihydrofluorescein; FL, fluorescein; L, lipophilic assay; PC, *β*-phycocyanin; PE, *β*-phycoerythrin; PGR, Pyrogallol Red.

**Table 5:** ORAC values of various food products and medicinal plants.

Material	Total Antioxidant Capacity
Açaí (*Euterpe oleracea*) berry, freeze-dried	1027 ^d^ (FL) [51]
Açaí fruit pulp	986 ^d^ (FL) [162], 4317 ^d^ (FL) [163]
Açaí juice, blend	17.6 ^b^ (T-FL) [164]
Açaí seed powder extract	31.5 ^d^ (FL) [165]
*Adenia chevalieri*, fruit	121 ^d^ (FL) [166]
Adzuki bean (*Vigna angularis*), flour	27.5 ^b^ (FL) [167]
Agave (*Agave* spp.), raw	12.5 ^b^ (T-FL) [23], 12.9 ^b^ (T-FL) [164]
Agave, cooked	29.4 ^b^ (T-FL) [23], 30.7 ^b^ (T-FL) [164]
Agave, dried	72.7 ^b^ (T-FL) [23], 75.2 ^b^ (T-FL) [164]
Agresto from Vermentino grapes	0.839 ^a^ (FL) [168]
Agresto from Sangiovese grapes	0.400 ^a^ (FL) [168]
Alexandrian laurel (*Calophyllum inophyllum*), fruit	110 ^d^ (FL) [166]
Alfalfa (*Medicago sativa*) sprouts	15.1 ^b^ (FL) [115]
Allspice (*Pimento dioica*)	0.441 ^b^ (FL) [169]
Almond (*Prunus dulcis*), seed	44.5 ^b^ (T-FL) [24]
Almonds, unpeeled	37.4 ^b^ (FL) [114]
Almonds, organic, unpeeled	53.1 ^b^ (FL) [114]
Aloe (*Aloe vera*)	1.88 ^b^ (PE) [170]
Alpine squill (*Scilla bifolia*), tepals	59.5 ^b^ (FL) [171]
Amaranth (*Amaranthus* spp.), leaves	92 ^d^ (FL) [103]
Amaranth, purple (*Amaranthus cruentus*)	28.9 ^b^ (PE) [170]
Amaranth flour	13.6 ^b^ (FL) [167]
Andean lupin (*Lupinus mutabilis*), seed	89.7 ^d^ (FL) [101]
Apple (*Malus domestica*)	13.8 ^b^ (FL) [172], 21.7 ^b^ (FL) [115],
Apple Braebum	20.6 ^b^ (FL) [114]
Apple Fuji, raw, unpeeled	26.8 ^b^ (FL) [114], 25.9 ^b^ (T-FL) [23]
Apple Gala, raw, unpeeled	28.3 ^b^ (FL) [51], 28.3 ^b^ (T-FL) [23,164]
Apple Golden Delicious	22.1 ^b^ (T-FL) [23], 26.4 ^b^ (FL) [117]
Apple Golden Delicious, raw, unpeeled	26.7 ^b^(T-FL) [164]
Apple Golden Delicious, peeled	22.1 ^b^(T-FL) [164]
Apple Golden Delicious, peel	40.0 ^b^ (FL) [115]
Apple Golden Delicious, pulp	7.07 ^b^ (FL) [115]
Apple Granny Smith, unpeeled	12.5 ^b^ (FL) [172], 35.2 ^b^ (FL) [114], 39.0 ^b^ (T-FL) [23]
Apple Red	39.2 ^b^ (FL) [114]
Apple Red, bulk, peeled	26.5 ^b^ (FL) [114]
Apple Red, bulk, unpeeled	36.1 ^b^ (FL) [114]
Apple Red Delicious, peeled	29.4 ^b^ (T-FL) [23]
Apple Red Delicious, unpeeled	42.8 ^b^ (T-FL) [23]
Apple Royal Deli	41.8 ^b^ (FL) [114]
Apple Royal G	27.3 ^b^ (FL) [114]
Apple Stark pulp	10.2 ^b^ (FL) [115]
Apple Stark peel	69.7 ^b^ (FL) [115]
Apple juice	4.08 ^b^ (FL) [21], 4.14 ^b^ (FL) [164], 4.3, 5.0 ^a^ (FL) [21], 22.2 (FL) ^a^, 31.78 (FL-EDTA) ^a^ [116], 8.9–14.3 ^a^ (PGR) [134]
Apple sauce	19.7 ^b^ (FL) [23]
Apricot (*Armeniaca vulgaris*), fruit	7.2 ^b^ (FL) [173], 10.6 ^b^ (FL) [115], 10.8 ^b^ (FL) [117], 82.7 ^d^ (FL) [166], 11.1 ^b^ (T-FL) [164], 13.4 ^b^ (T-FL) [23]
Apricot fruit, bulk, unpeeled	31.6 ^b^ (FL) [114]
Apricot fruit, bulk, peeled	18.4 ^b^ (FL) [114]
Apricot juice	15.5 ^a^ (PGR) [134]
Artichoke (*Cynara cardunculus*)	65.5 ^b^ (FL) [98], 92.8 ^b^ (T-FL) [23]
Artichoke hearts	65.5 ^b^ (FL) [51]
Artichokes, Ocean Mist, boiled	94.2 ^b^ (T-FL) [164]
Artichokes, Ocean Mist, Microwaved	94.0 ^b^ (T-FL) [164]
Asian pear (*Pyrus pyrifolia*), unpeeled	22.5 ^b^ (FL) [114]
Asian pear peeled	8.57 ^b^ (FL) [114], 2.32 ^b^ (T-FL) [174]
Asparagus (*Asparagus officinalis*), raw	11.4 ^b^ (FL) [175], 12.9 ^b^ (FL) [98], 11.5 ^b^ (T-FL) [174], 22.5 ^b^ (T-FL) [164], 29.2 ^b^ (T-FL) [23]
Asparagus, white, raw	2.96 ^b^ (FL) [164]
Asparagus, cooked	16.4 ^b^ (FL) [23]
Assidat zgougou (Tunisian dish)	1065 ^d^ (FL) [176]
Athlete supplement, Active Edge Blueberry	222 ^a^ (FL) [89]
Athlete supplement, Active Edge Capsule 2 × 435 mg capsules	173 ^d^ (FL) [89]
Athlete supplement, Active Edge Cherry Bottle	247 ^a^ (FL) [89]
Athlete supplement, Active Edge Cherry Sachet	201 ^a^ (FL) [89]
Athlete supplement, Active Edge Pomegranate	96.6 ^a^ (FL) [89]
Athlete supplement, CurraNZ 1 × 300 mg capsule	3868 ^d^ (FL) [89]
Athlete supplement, Healthspan Elite Sour Cherry	297 ^a^ (FL) [89]
Athlete supplement, PAS Cherry Bomb	209 ^a^ (FL) [89]
Athlete supplement, POM Wonderful	23.8 ^a^ (FL) [89]
Athlete supplement, SIS Rego Cherry Juice	336 ^a^ (FL) [89]
Avocado (*Persea americana*)	19.2 ^b^ (T-FL) [164], 19.3 ^b^ (T-FL) [24], 79.0 ^b^ (T-FL) [24]
Avocado, Bacon	13.9 ^b^ (FL) [114]
Avocado, Edranol	7.79 ^b^ (FL) [114]
Avocado, Ester	17.9 ^b^ (FL) [114]
Avocado Fuerte	13.9 ^b^ (FL) [114]
Avocado, Hass	48.5 ^b^ (FL) [114], 19.3 ^b^ (T-FL) [23]
Avocado Negra TLC	16.1 ^b^ (FL) [114]
Baby food, apple	48.2 ^b^ (T-FL) [164]
Baby food, apple sauce (Gerber)	41.2 ^b^ (FL) [23]
Baby food, apple blueberry (Gerber)	48.2 ^b^ (FL) [23]
Baby food, banana (Gerber)	26.6 ^b^ (FL) [23]
Baby food, peach (Heinz)	62.6 ^b^ (FL) [23]
Baby food, pear (Gerber)	25.5 ^b^ (FL) [23]
Baby food, pear juice	4.1 ^b^ (FL) [164]
Balsam pear (*Momordica charantia*)	3.43 ^b^ (PE) [170]
Banana (*Musa* spp.), fruit, raw	8.0 ^b^ (T-FL) [164], 8.8 ^b^ (T-FL) [23]
Banana, Nam-wa variety	2.6 ^b^ (FL) [177]
Basil (*Ocymum basilicum*), raw	48.1 ^b^ (FL) [98]
Basil, dried	611 ^d^ (FL) [51]
Basil leaves, dried	675.5 ^d^ (T-FL) [23]
Basil, ‘Medinette’ leaves, dried	402 ^d^ (FL) [178], 96.2 ^d^ (T-FL) [92]
Basil, Indian, leaves, dried	157.7 ^d^ (T-FL) [92]
Basil, ‘Persian’ leaves, dried	96.8 ^d^ (T-FL) [92]
Bastilla, mini chicken (Moroccan dish)	1111 ^d^ (FL) [176]
Bean, common (*Phaseolus vulgaris*)	4.30 ^b^ (FL) [175], 62.6 ^b^ (FL) [179]
Bean, black	79.3 ^b^ (FL) [180]
Bean, black, mature seeds, raw	80.4 ^b^ (T-FL) [23], 86.1 ^b^ (T-FL) [164]
Bean, black, mature seeds, boiled	22.5 ^b^ (FL) [164]
Bean, black turtle soup, mature seeds, raw	64.2 ^b^ (FL) [164], 80.4 ^b^ (FL) [51]
Bean, black, flour	40.3 ^b^ (FL) [167]
Bean, green	7.45 ^b^ (FL) [117], 14.5 ^b^ (FL) [181]
Bean, lima canned	2.43 ^b^ (T-FL) [23]
Bean, navy, mature seeds, raw	18.6 ^b^ (T-FL) [164]
Bean, navy, dry mature	24.7 ^b^ (T-FL) [23]
Bean, pink, mature seeds, raw	83.2 ^b^ (FL) [164]
Bean, pinto, mature seeds, dry	123.6 ^b^ (T-FL) [23]
Bean, pinto, mature seeds, raw	80.3 ^b^ (T-FL) [164]
Bean, pinto, mature seeds, boiled	9.04 ^b^ (T-FL) [164]
Bean, red kidney, dry mature	61.2 ^b^ (FL) [180], 86.1 ^b^ (FL) [51], 144.1 ^b^ (T-FL) [23]
Bean, red kidney, flour	34.6 ^b^ (FL) [167]
Bean, small red, dry mature	149.2 ^b^ (T-FL) [23]
Bean, snap raw	2.67 ^b^ (T-FL) [23]
Bean, snap canned	2.90 ^b^ (T-FL) [23]
Bean (*Vigna radiata f. aureus*) sprout	16.1 ^b^ (FL) [175]
Beef, longissimus muscle	31.9 ^d^ (FL), 1.29 ^d^ (L-FL) [90]
Beef, raw and cooked	1.30 ^b^ and 1.58 ^b^ (L-FL) [90]
Beef steak, raw and broiled	28.1 and 33.0 ^b^ (FL) [90]
Beef stew, French dish	371.1 ^d^ (FL) [176]
Beer, ale, Belgian	1.12–1.65 ^a^ (FL) [88]
Beer, bright lager	4.61–5.92 ^a^ (FL) [158]
Beer, dry-hoped	11.5 ^b^ (FL) [182]
Beer, India Pale Ale	23.0 ^a^ (FL) [183]
Beer, lager	10.2 ^b^ (FL) [182]
Beer, lager, Portuguese	3.10–10.7 ^a^ (FL) [88]
Beer, low alcoholic	2.01–7.91 ^b^ (FL) [182]
Beer, Premium lager	3.70 ^a^ (FL) [183]
Beer, Stout	26.7 ^a^ (FL) [183]
Beer, Trappist brown	12.3 ^b^ (FL) [182]
Beet (*Beta vulgaris*, var. *rubra*), raw	12.2 ^b^ (FL) [175], 12.6 ^b^ (FL) [181], 26.2 ^b^ (FL) [115], 27.2 ^b^ (FL) [98], 17.9 ^b^ (T-FL) [164], 19.5 ^b^ (T-FL) [164], 27.7 ^b^ (T-FL) [23]
Beet, green, frozen	11.7 ^b^ (FL) [115]
Bilberry (*Vaccinium myrtillus*)	767 ^d^ (FL) [162]
Bilberry juice	12.51 (PE), 34.66 (FL) ^a^ [19]
Birch (*Betula pendula*), leaves	1185 ^d^ (FL) [178]
Bissara, Moroccan dish	128.4 ^d^ (FL) [176]
Bitter melon (*Momordica charantia*)	6.90 ^b^ (T-FL) [174]
Black cherry (*Prunus serotina*), fruit	26.8 ^d^ (FL) [101]
Blackberry (*Rubus fruticosus*)	14.1 ^d^ (FL) [184], 20.4 ^b^ (FL) [185], 59.1 ^b^ (FL) [51], 53.5 ^b^ (T-FL) [23], 59.1 ^b^ (T-FL) [164], 32.7 ^b^ (PGR) [73]
Blackberry leaves	1806 ^d^ (FL) [186]
Blackberry, organic	423 ^d^ (FL) [162]
Blackberry (*Rubus caesius*)	74.2 ^b^ (FL) [101]
Blackberry, evergreen (*Rubus laciniatus*)	27.5 ^b^ (PE) [187]
Blackberry (*Morus nigra*) fruit	63.7 ^b^ (FL) [173]
Blackthorn (*Prunus spinosa*)	79.1 ^b^ (FL) [101], 68.8 ^b^ and 89.4 ^b^ (FL) [96]
Blue Candle cactus (*Myrtillocactus geometrizans*), fruit	69.8 ^b^ (FL) [188]
Blue corn meal	6.84 ^b^ (FL) [23]
Blueberry (*Vaccinium* spp.)	35.9 ^b^ and 37.3 ^b^ (PE) [85], 22.3 ^b^ FL [185], 46.7 ^b^ (FL) [51], 54.8 ^b^ (FL) [114], 56.1 ^b^ (FL) [172], 98.8 ^b^ (FL) [101], 509 ^d^, 880 ^d^ (FL) [162], 46.7 ^b^ (T-FL) [164], 62.2 ^b^ (T-FL) [24], 17.4 **^b^** (PGR) [73]
Blueberry, Aurora	68.8 ^b^ (FL) [114]
Blueberry, Bluecrop	71.3 ^b^ (FL) [114]
Blueberry, Bluegold	87.6 ^b^ (FL) [114]
Blueberry, Brigitta	55.4 ^b^ (FL) [114]
Blueberry, Duke	48.6 ^b^ (FL) [114]
Blueberry, Elliot	88.7 ^b^ (FL) [114]
Blueberry, juice	7.51 (PE), 23.6 ^b^ (FL) [164], 23.8 (FL)^a^ [19], 29.1 ^b^ (FL) [21], 32.7 ^a^ (FL) [21]
Blueberry, lowbush	92.6 ^b^ (T-FL) [23]
Blueberry, nectar	1.78 ^a^ (FL) [138]
Blueberry, organic	618 ^d^ (FL) [162]
Blueberry, wild	96.2 **^b^** (FL) [51]
Boldo (*Peumus boldus*), extract	2728 ^c^ (FL), 53 ^c^ (PGR) [72]
Boysenberry (*Rubus fruticosus x idaeus*)	42.2 ^b^ (PE) [187], 216 ^d^ (FL) [164]
Bread, butternut all whole grain wheat, Chicago Baking Co	21.0 ^b^ (T-FL) [23]
Bread, Multi-Grain	14.2 ^b^ (T-FL) [164]
Bread, oatnut	12.2 ^b^ (FL) [117]
Bread, Oatnut, Brownberry	13.2 ^b^ (T-FL) [23]
Bread, pumpernickel, Brownberry	19.6 ^b^ (T-FL) [23]
Bread, whole grain Healthy Choice	14.2 ^b^ (T-FL) [23]
Breakfast cereals, corn flakes, ready to eat	23.0 ^b^ (FL) [117]
Breakfast cereals, granola with raisins, low-fat, Kellog’s, ready to eat	22.9 ^b^ (T-FL) [23]
Breakfast cereals, Life, Quaker, ready to eat	15.2 ^b^ (T-FL) [23]
Breakfast cereals, Original Shredded Wheat, Post, ready to eat,	13.0 ^b^ (T-FL) [23]
Breakfast cereals, oat bran, Quaker, ready to eat	18.9 ^b^ (T-FL) [23]
Breakfast cereals, oat, instant, fortified, plain, dry	23.1 ^b^ (T-FL) [164]
Breakfast cereals, oat bran, Quaker, uncooked, hot	24.8 ^b^ (T-FL) [23]
Breakfast cereals, oat, old-fashioned, Quaker, uncooked	17.1 ^b^ (T-FL) [23]
Breakfast cereals, oat, quick, 1-min, Quaker, uncooked	21.7 ^b^ (T-FL) [23]
Breakfast cereals, oatmeal, instant, Quaker, uncooked	23.1 ^b^ (T-FL) [23]
Breakfast cereals, squares toasted oatmeal, Quaker, ready to eat	21.4 ^b^ (T-FL) [23]
Breakfast cereals, Total, General Mills	23.6 ^b^ (T-FL) [23]
Breakfast cereals, wheat, shredded, plain, sugar and salt free, ready-to-eat	13.03 ^b^ (T-FL) [164]
Brier (*Rosa canina*)	201.1 ^b^ (FL) [101]
Broad bean (*Vicia faba*), seed	29.5 ^d^ (FL) [101]
Broccoli (*Brassica oleracea* var. *italica*), *raw*	11.8 ^b^ (FL) [177], 16.1 ^b^ (FL) [181], 35.3 ^b^ (FL) [115], 35.3 ^b^ (FL) [98], 37.0 ^d^ (L-FL) [135], 15.9 ^b^ (T-FL) [23,24], 15.1 ^b^ (T-FL) [164]
Broccoli, boiled	21.6 ^b^ (T-FL) [164]
Broccoli cooked	12.6 ^b^ (T-FL) [23]
Broccoli, frozen	4.96 ^b^ (FL) [115]
Broccoli, raab cooked	15.6 ^b^ (T-FL) [23], 15.9 ^b^ (T-FL) [164]
Broccoli, raab raw	30.8 ^b^ (T-FL) [23]
Buckwheat, common (*Polygonum fagopyrum*), flour	17.9–109.1 ^b^ (FL) [99]
Buckwheat, tartary (*Fagopyrum esculentum*), flour	81.8 ^b^ (FL) [167], 555.8 ^d^ (FL) [99]
*Buddleia globosa* extract	2175 ^c^ (FL), 25 ^c^ (PGR) [72],
Burdock, greater (*Arctium lappa*), roots	365 ^d^ (FL) [178], 67.5 ^b^ (T-FL) [174]
Cabbage (*Brassica oleracea*), green, raw	5.1 ^b^ (FL) [189], 6.43 ^b^ (FL) [175], 5.29 ^b^ (T-FL) [164], 8.56 ^b^ (FL), [98], 13.6 ^b^ (T-FL) [23]
Cabbage, green, savoy (cv Sabauda), raw	20.5 ^b^ (FL) [98]
Cabbage, green, savoy (cv Sabauda), boiled	20.5 ^b^ (FL) [164]
Cabbage, boiled	8.56 ^b^ (T-FL) [164]
Cabbage, Chinese celery (*Brassica pekinensis* var. *cylindrica*)	6.19 ^b^ (FL) [175]
Cabbage, red (*Brassica oleracea* var. *capitata f*. *rubra*), raw	23.1 ^b^ (FL) [175], 110.3 ^b^ (FL) [189], 22.5 ^b^ (T-FL) [23], 25.0 ^b^ (T-FL) [164], 34.7 ^b^ (T-FL) [174]
Cabbage, red, cooked	31.5 ^b^ (FL) [23]
Cactus pear (*Opuntia* spp.), fruit pulp/juice, green orange red purple	3.68 ^b^/5.45 ^a^ (PE) [190] 4.36 ^b^/5.83 ^a^ (PE) [190] 4.44 ^b^/6.35 ^a^ (PE) [190] 8.16 ^b^/11.2 ^a^ (PE) [190]
Calabash (*Crescentia cujete*) fruit	11.8^b^ (FL) [108]
Calafate (*Berberis microphylla*) berry	256.6 ^b^ (FL) [114], 467.5 ^d^ (FL) [191]
Caltrop (*Tribulus terrestris*), aerial parts	819 ^d^ (FL) [178]
Cambuci (*Campomanesia phaea*) fruit	4.57–11.3 ^b^ (FL) [120]
Candies, chocolate, dark	208.2 ^b^ (T-FL) [164]
Candies, milk chocolate	75.2 ^b^ (T-FL) [164]
Candies, semisweet chocolate	180.5 ^b^ (T-FL) [164]
Cantaloupe (*Cucumis melo*)	3.1 ^b^ (T-FL) [24]
Carambola (*Averrhoa carambola*)	12.9 ^b^ (FL) [192]
Caraway (*Carum carvi*)	10.7 ^b^ (PE) [170]
Cardamom (*Elettaria cardamomum*)	27.6 ^b^ (FL) [98], 56 ^b^ (FL) [182]
Carob (*Ceratonia siliqua*) beverage	1.9 ^a^ (FL) [193]
Carrot (*Daucus carota* var. *sativa*), raw	1.07 ^b^ (FL) [98], 2.47 ^b^ (FL) [175], 2.71 ^b^ (FL) [115], 3.3 ^b^ (FL) [172], 4.8 ^b^ (FL) [181], 4.07 ^b^ (T-FL) [174], 6.96 ^b^ (FL) [115], 8.13 ^d^ (L-FL) [135], 6.97 ^b^(T-FL) [164], 12.2 ^b^ (T-FL) [23]
Carrot, baby, raw	4.36 ^b^ (T-FL) [23,24]
Carrot, cooked	4.99 ^b^ (FL) [115], 3.26 ^b^ (T-FL) [164], 3.71 ^b^ (T-FL) [23]
Carrot steamed	2.63 ^b^ (FL) [115]
Cashew (*Anacardium occidentale*)	7.8 ^b^ (FL) [108], 19.5 ^b^ (T-FL) [164], 20.0 ^b^ (T-FL) [23,24]
Cassava (*Manihot esculenta*), leaves	470 ^d^ (FL) [103]
Cauchao (*Amomyrtus luma*) berry	451.1 ^d^ (FL) [194]
Cauliflower (*Brassica oleracea* var. *botrytis*), raw	8.28 ^b^ (FL) [175], 9.25 ^b^ (FL) [115], 9.25 ^b^ (FL) [98], 6.47 ^b^ (T-FL) [23], 8.70 ^b^ (T-FL) [164]
Cauliflower, boiled	7.39 ^b^ (FL) [164]
Cauliflower, frozen	6.20 ^b^ (T-FL) [164]
Cauliflower, green, raw	1.36 ^b^ (T-FL) [164]
Cauliflower, green, cooked	13.9 ^b^ (T-FL) [164]
Cauliflower, purple, raw	20.8 ^b^(T-FL) [164]
Cauliflower, purple, cooked	22.1 ^b^ (T-FL) [164]
Cauliflower, steamed	6.20 ^b^ (FL) [115]
Celery (*Apium graveolens*)	3.43 ^b^ (FL) [98], 8.37 ^b^ (FL) [175], 5.52 ^b^ (T-FL) [164], 5.74 ^b^ (T-FL) [23,24]
Celery, leaves	113.5 ^b^ (FL) [181]
Celery, root	15.3 ^b^ (FL) [181]
Century plant (*Agave americana*), leaf	70.3 ^d^ (FL) [101]
Chamomile (*Matricaria chamomilla*), flowers	814 ^d^ (FL) [178]
*Chenopodium ambrosioides* extract	395 ^c^ (FL) [72], 3.3 ^c^ (PGR) [72]
Chia (*Salvia hispanica*) seeds	517.3 ^b^ (FL), 6.48 ^b^ (L-FL) [195]
Chinese hawthorn (*Crataegus pinnatifida*)	1240 ^d^ (FL) [91]
Chinese sage (*Salvia miltiorrhiza*), herb	1320 ^d^ (FL) [91]
Cherry (*Prunus avium*)	25.8 ^b^ (FL) [101], 37.5 ^b^ (FL) [51], 202 ^d^ (FL) [162], 33.6 ^b^ (T-FL) [23]
Cherry, Bing	61.2 ^b^ (FL) [114]
Cherry, Brooks	55.7 ^b^ (FL) [114]
Cherry, Granel	39.2 ^b^ (FL) [114]
Cherry, Lapins	38.5 ^b^ (FL) [114]
Cherry, Rainier	42.3 ^b^ (FL) [114]
Cherry, Tart	149 ^d^ (FL) [162]
Cherry, Van	37.3 ^b^ (FL) [114]
Cherry, black, juice	23.7 ^b^(FL) [164]
Chicken, roasted	13.6 ^b^ (FL) [117]
Chicken Blood Vine (*Spatholobus suberectus*), herb	1990 ^d^ (FL) [91]
Chickpea (*Cicer arietinum*) mature seeds, raw	8.47 ^b^ (FL) [164], 9.26 ^b^ (FL) [179], 34.7 ^b^ (FL) [180]
Chickpea (black) flour	17.1 ^b^ (FL) [167]
Chickpea, fruit	104 ^d^ (FL) [166]
Chicory (*Cichorium intybus*), aerial parts	398 ^d^ (FL) [178]
Chilchen (Red Berry Beverage)	7.40 ^b^ (FL) [23]
Chilean blackcurrant (*Ribes magellanicum*)	360 ^d^ (FL) [196]
Chilean strawberry (*Fragaria chiloensis*)	313.1 ^d^ (FL) [191]
Chili (*Capsicum annuum*) green	5.34 ^b^ (FL) [98], 18.4 ^b^ (FL) [175]
Chili, red	5.09 ^b^ (FL) [98], 27.8 ^b^ (FL) [175]
Chili powder	236.4 ^b^ (T-FL) [23]
Chinese angelica (*Angelica sinensis*), root	80.7 ^d^ (FL) [166]
Chinese figwort (*Scrophularia ningpoensis*)	77 ^d^ (FL) [91]
Chinese foxglove (*Rehmannia glutinosa*)	65 ^d^ (FL) [91]
Chinese ground orchid (*Bletilla striata*)	100 ^d^ (FL) [91]
Chinese honey locust (*Gleditsia sinensis*)	570 ^d^ (FL) [91]
Chinese lantern (bladder cherry) (*Physalis alkekengi*)	135 ^d^ (FL) [166]
Chinese mugwort (*Artemisia argyi*)	1150 ^d^ (FL) [91]
Chinese peony (*Paeonia lactiflora*)	350 ^d^ and 680 ^d^ (FL) [91]
Chinese rhubarb (*Rheum officinale*), root	79.3 ^d^ (FL) [166]
Chips, Olestra tortilla	15.5 ^b^ (T-FL) [164], 17.0 ^b^ (T-FL) [23]
Chips, tortilla, low fat, made with olestra, nacho cheese	18.6 ^b^ (T-FL) [23], 15.5 ^b^ (T-FL) [164]
Chitosan (50–190 kDa)	0.02 ^b^ (FL) [197]
Chives *Allium schoenoprasum*	9.15 ^b^ (PE) [170], 20.9 ^b^ (FL) [98]
Chocolate, baking	1039.7 ^b^ (T-FL) [23]
Chocolate, baking chips	202 ^b^ (T-FL) [198]
Chocolate, baking unsweetened, squares	499.4 ^b^ (T-FL) [164]
Chocolate, dark	227 ^b^ (T-FL) [198]
Chocolate, dutched powder	402 ^b^ (T-FL) [198]
Chocolate, milk	80 ^b^ (T-FL) [198]
Chocolate, milk, candy bar	81.7 ^b^ (T-FL) [23]
Chocolate, natural powder	826 ^b^ (T-FL) [198]
Chocolate, syrup	63.3 ^b^ (FL) [164]
Chocolate, unsweetened	496 ^b^ (T-FL) [198]
Chokeberry (*Aronia melanocarpa*), fruit	160.8 ^b^ (FL) [101], 2140 ^d^ (FL) [199], 160.6 ^b^ (T-FL) [200]
Chokeberry juice concentrate	418 ^a^ (FL) [162]
Chokeberry, leaves	1363 ^d^ (FL) [186]
Chorba, white (Algerian dish)	391.4 ^d^ (FL) [176]
Chupón (*Greigia sphacelata*) fruit	35.3 ^b^ (PGR) [201]
Cinnamon (*Cinnamomum verum*), bark	22.1 ^b^ (FL) [202], 907 ^b^ (FL) [182]
Cinnamon, leaves	44.8 ^b^ (FL) [202]
Cinnamon, ground	1256 ^b^ (FL) [144], 1314 ^b^ (FL) [51], 2675.4 ^b^ (T-FL) [23]
Cinnamon essential oil	7.71 ^b^ (FL) [130]
Clove (*Syzygium aromaticum*), ground	2903 ^b^ (FL) [51], 3144.5 ^b^ (T-FL) [23]
Clove essential oil	10.6 ^b^ (FL) [130]
Cocoa, dry powder	4.85 ^b^ (FL) [117,164], 556.5 ^b^ (FL) [51]
Coffee (*Coffea arabica*), arabica, conventional	290.0 ^b^ (FL) [203]
Coffee, arabica, pulp dried powder	8400–12,000 ^d^ (PE) [83]
Coffee, cascara	216–614 ^d^ (FL) [104]
Coffee, elephant	250.9 ^b^ (FL) [203]
Coffee, extract	9.59 ^a^ (FL) [138]
Coffee, powder	735 and 823 ^d^ (FL) [204], 10,400 (FL)^d^ and 12,800 (FL)^d^ [18]
Cogon grass (*Imperata cylindrica*)	130 ^d^ (FL) [91]
Cookie, oatmeal raisin, Pepperidge Farm	20.0 ^b^ (T-FL) [23]
Coriander (*Coriandrum sativum*)	273 ^b^ (FL) [182]
Coriander leaves, raw	51.4 ^b^ (FL) [98], 60.5 ^b^ (FL) [175]
Corn (*Zea mays*), raw	10.4 ^b^ (FL) [175], 7.28 ^b^ (T-FL) [23], 7.6 and 9.2 ^b^ (T-FL) [174]
Corn, sweet yellow, canned	4.13 ^b^ (T-FL) [23]
Corn, sweet, yellow, frozen	5.22 ^b^ (T-FL) [23]
Cornelian cherry (*Cornus mas*)	49.0 ^b^ (FL) [101], 119.2 ^b^ and 175.9 ^b^ (FL) [96]
Cotton, Levant (*Gossypium herbaceum*), root	122 ^d^ (FL) [166]
Couscous with meat and vegetables (Tunisian dish)	431.2 ^d^ (FL) [176]
Courgette (*Cucurbita pepo*)	1.80 ^b^ and 3.44 ^b^ (FL) [98]
Cowherb (*Vaccaria segetalis*)	200 ^d^ (FL) [91]
Cowpea (*Vigna sinensis*)	20.1 ^b^ (FL) [175]
Cowpea (*Vigna unguiculata*), mature	43.4 ^b^ (T-FL) [164]
Cranberry (*Vaccinium oxycoccos*)	70.0 ^b^ (FL) [101], 90.9 ^b^ (FL) [51], 94.6 ^b^ (T-FL) [23]
Cranberry juice	14.5 ^b^ (FL) [164], 2.8 ^a^ (PGR) [134]
Cranberry juice, red	8.65 ^b^ (FL) [164]
Cranberry juice, white	2.32 ^b^ (FL) [164]
Creeping thistle (*Cirsium setosum*)	370 ^d^ (FL) [91]
Cucumber (*Cucumis sativus*), unpeeled	1.2 ^b^ (FL) [181], 1.82, 2.51 ^b^ (FL) [175], 2.52 ^b^ (FL) [98], 1.15 ^b^ (T-FL) [23], 2.32 ^b^ (T-FL) [164], 2.72 ^b^ (T-FL) [174]
Cucumber, peeled	1.23 ^b^ (T-FL) [23], 1.40 ^b^ (T-FL) [164]
Cumin (*Cuminum cyminum*)	768.0 ^b^ (FL) [98]
Cumin fruit	125 ^d^ (FL) [166]
Cumin seed	504 ^b^ (FL) [51]
Curly dock (*Rumex crispus*), leaves	298.0 ^d^ (FL) [101]
Currant, black (*Ribes nigrum*)	96.0 ^b^ (FL) [101], 100.6 ^b^ (FL) [200], 148 ^d^ (FL) [166], 389 ^d^ (FL) [162], 79.6 ^b^ (T-FL) [164]
Curry (*Bergera koenigii*), powder	485 ^b^ (T-FL) [23]
Cyathula (*Cyathula officinalis*), root	43 ^d^ (FL) [91]
Dandelion (*Taraxacum officinale*), aerial parts	2.35 ^b^ (PE) [170], 381 ^d^ (FL) [178]
Dates (*Phoenix dactylifera*), Deglet Noor, dried	39.0 ^b^ (T-FL) [23]
Dates, Medjool	23.9 ^b^ (T-FL) [23]
Dendrobium, Noble (*Dendrobium nobile*), fruit	120 ^d^ (FL) [166]
Dill (*Anethum graveolens*)	29.1 ^b^ (PE) [170], 10.5 ^b^ (FL) [181], 43.9 ^b^ (FL) [98]
Diverse wormwood (*Artemisia anomala*) herb	1400 ^d^ (FL) [91]
Durian (*Durio zibethinus*), fruit	(360.9–377.6)^d^ (FL) [205]
Egg, chicken (*Gallus gallus domesticus*) yolk, raw	147.1 ^d^ (FL) [206]
Egg, chicken yolk, boiled	74.8 ^d^ (FL) [206]
Egg, chicken yolk, fried	83.5 ^d^ (FL) [206]
Eggplant (*Solanum melongena*), raw	3.44 ^b^ (FL) [115], 6.7 ^b^ (FL) [172], 9.32 ^b^ (FL) [164], 12.8 ^b^ (FL) [175], 16.2 ^b^ (FL) [181], 18.5 ^b^ and 21.7 ^b^ (T-FL) [174], 25.3 ^b^ (T-FL) [23]
Eggplant, Black beauty	11.9 ^b^ (FL) [98]
Eggplant, Violetta lunga	14.1 ^b^ (FL) [98]
Eggplant, boiled	2.45 ^b^ (FL) [164]
Eggplant, frozen	0.37 ^b^ (FL) [115]
Eggplant slices, raw	3.81 ^b^ (FL) [115]
Eggplant slices, steamed	2.45 ^b^ (FL) [115]
Elderberry (*Sambucus nigra*), fruit	205.4 ^b^ (FL) [101], 953 ^d^ (FL) [162], 147.0 ^b^ (T-FL) [200]
Endive (*Cichorium endivia* ssp. *Endivia*)	10.3 ^b^ (FL) [175]
Female ginseng (*Angelica sinensis*)	78 ^d^ (FL) [91]
Fennel (*Foeniculum vulgare*)	3.61 ^b^ (FL) [98]
Fennel, bulb, raw	3.07 ^b^ (FL) [164], 5.88 ^b^ (PE) [170]
Fenugreek (*Trigonella foenum-graecum*), fruit	75.2 ^d^ (FL) [166]
Fenugreek, seeds	327 ^d^ (FL) [178]
Feverfew (*Tanacetum parthenium*)	10.1 ^b^ (PE) [170]
Fig (*Ficus carica*), raw	13.6 ^b^ (FL) [101], 11.0 ^b^ (T-FL) [174], 33.8 ^b^ (T-FL) [23]
Fish stew (French dish)	1239 ^d^ (FL) [176]
Fortune’s Drynaria (*Drynaria fortunei*), rhizome	700 ^d^ (FL) [91]
Frankincense (*Boswellia carterii*)	49 ^d^ (FL) [91]
Garlic (*Allium sativum*), raw	19.4 ^b^ (FL) [80], 22.4 ^b^ (FL) [175], 26.1 ^b^ (FL) [207], 53.5 ^b^ (FL) [98], 23.1 ^b^ (T-FL) [174], 57.1 ^b^ (T-FL) [164]
Garlic, purple	67.8 ^b^ (FL) [117]
Garlic powder	66.7 ^b^ (T-FL) [23]
Geranium, rose (*Pelargonium graveolens*)	38.8 ^b^ (PE) [170]
Ginger (*Zingiber officinalis*), root, fresh	31.6 ^b^ (FL) [175], 148.4 ^b^ (FL) [98], 187.4 ^b^ (FL) [110], 721 ^d^ (FL) [182], 22.0 and 27.9 ^b^ (T-FL) [174]
Ginger, ground	288.1 ^b^ (T-FL) [23]
Goji berry (Wolfberry) (*Lycium barbarum*)	32.9 ^b^ (T-FL) [164], 1377 ^d^ (FL) [208]
Goji berry, black (*Lycium ruthenicum*)	141 ^d^ (FL) [166]
Gooseberry (*Ribes uva-crispa*)	35.8 ^b^ (FL) [200], 33.3 ^b^ (T-FL) [164]
Goosefoot, oak-leaved (*Chenopodium glaucum*), leaves	104 ^d^ (FL) [166]
Gourd, bitter (*Momordica charantia*)	5.25 ^b^ (FL) [175]
Gourd, hairy (*Benincasa hispida* var. *chiehqua*)	2.02 ^b^ (FL) [175]
Gourd, winter (*Benincasa hispida*)	1.58 ^b^ (FL) [175]
Grape (*Vitis vinifera*), black	17.5 ^b^(FL) [164]
Grape, black, table, 9 new Apulian genotypes	349.5–601.5 mmol/kg skin (FL) [209]
Grape, red	12.6 ^b^ (FL) [23], 18.4 ^b^(FL) [164], 26.8 ^b^ (FL) [101]
Grape, red, juice (market)	7.81 ^a^ (FL), 10.91 ^a^ (FL-EDTA) [116]
Grape white or green	6.3 ^b^ (FL) [101], 10.2 ^b^(FL) [164]
Grape juice	12.1 ^a^ (PE) and 31.4 (FL)^a^ [19], 32.6 ^a^, 33.7 ^a^ (FL) [18]
Grape, Gizil Ozoum	4726.8 ^d^ (FL) [133]
Grape, Monastrell	43.6 ^a^ (FL) [210]
Grape, Rezaie	3543.4 ^d^ (FL) [133]
Grape, Shani-A	2735.9 ^d^ (FL) [133]
Grape, Shirazi-A	3821.9 ^d^ (FL) [133]
Grape, Shirazi-B	3452.9 ^d^ (FL) [133]
Grape (red) juice	19.1 ^a^ (FL) [21]
Grape (white) juice	3.4, 12.4 ^a^ (FL) [21]
Grape (Concord) juice	20.0 ^a^ (FL) [21], 23.9 ^b^ (FL) [164]
Grape nectar	2.70 ^a^ (FL) [138]
Grapefruit (*Citrus* × *paradisi*), red	16.1 ^b^ (FL) [211], 15.5 ^b^ (T-FL) [23]
Grapefruit, white	14.5 ^b^ and 15.0 ^b^ (FL) [211]
Grapefruit juice	12.9 ^a^ (FL) [23]
Grapefruit juice, white raw	12.4 ^b^(FL) [164]
Great burnet (*Sanguisorba officinalis*), root	1940 ^d^ (FL) [91]
Green silk turmeric (*Curcuma phaeocaulis*)	170 ^d^ (FL) [91]
Gulasch, German dish	624.9 ^d^ (FL) [176]
Guava (*Psidium guajava*) fruit	18.4 ^b^ (FL) [177], 16.7 ^b^ (FL) [192]
Guava, Fan Retief	18.2–25.5 ^b^ (FL) [212]
Guava, red-fleshed	19.9 ^b^ (T-FL) [164]
Guava, white-fleshed	9.9 ^b^ (FL) [192], 25.2 ^b^ (T-FL) [164]
Guava Allahabad Safeda	25.5 ^b^ (FL) [212]
Guava Ruby Supreme	18.2 ^b^ (FL) [212]
Gumbo (*Abelmoschus esculentus*)	14.6 ^b^ (FL) [181]
Hairy agrimony (*Agrimonia pilosa*), herb	1440 ^d^ (FL) [91]
Ham, cured	33.6 ^b^ (FL) [117]
*Haplopappus baylahuen* extract	2250 ^c^ (FL), 47 ^c^ (PGR) [72]
Hardy kiwi (*Actinidia arguta*)	94.2 ^b^ (T-FL) [174]
Harira, Moroccan dish	1052.7 ^d^ (FL) [176]
Hawthorn (*Crataegus monogyna*), flowers + leaves	2163 ^d^ (FL) [178]
Hawthorn, leaves	1405 ^d^ (FL) [186]
Hawthorn (*Crataegus mollis*)	153.6 ^b^ (FL) [101]
Hazelnuts (*Corylus avellana*)	96.5 ^b^ (T-FL) [23]
He Shou Wu (*Polygonum multiflorum*)	790 ^d^ (FL) [91]
Hibiscus (*Hibiscus rosa-sinensis)*	477 ^b^ (FL) [182]
Hazelnut (*Corylus avellana*) shells	3100–3282 ^b^ (FL) [213]
Hogweed (*Heracleum scabridum*), root	104 ^d^ (FL) [166]
Honey, acacia	2.12 ^b^ (PE) [82], 3.00 ^b^ (PE) [214]
Honey, buckwheat	9.75 ^b^ (PE) [214], 11.6 ^b^ (PE) [82]
Honey, cardoon	42.8 ^b^ (FL) [215]
Honey, chestnut	8.90 ^b^ (PE) [82]; 20.2 ^b^ (FL) [216]
Honey, clover	2.15 ^b^ (PE) [82], 6.34 ^b^ (PE) [214]
Honey, eucalyptus	7.40 ^b^ (FL) [215]
Honey, fireweed	3.09 ^b^ (PE) [214]
Honey, goldenrod	15.4 ^b^ (FL) [216]
Honey, honeydew	6.30 ^b^ (PE) [82]
Honey, linden	44.3 ^b^ (FL) [216]
Honey, milkweed	12.6 ^b^ (FL) [216]
Honey, soy	8.34 ^b^ (PE) [214]
Honey, strawberry tree	21.1 ^b^ (PE) [82], 39.6 ^b^ (FL) [215]
Honey, sunflower	0.41 ^b^ (FL) [215]
Honey, thyme	10.6 ^b^ (FL) [215]
Honey, tupelo	6.48 ^b^ (PE) [214]
Honeydew melon (*Cucumis melo inodorus*)	2.4 ^b^ (T-FL) [24], 2.3 ^b^ (FL) [101]
Honeysuckle, Dasystyle (*Lonicera dasystyla*), root	103 ^d^ (FL) [166]
Hop (*Humulus lupulus*), flowers	749 ^d^ (FL) [178]
Hyacinth (*Hyacintus orientalis*), tepals	139.0 ^b^ (FL) [171]
Hyssop (*Hyssopus officinalis*)	60.5 ^b^ (FL) [98]
Ice-cream bean (*Inga edulis*)	Fruit 17.5 ^b^; Leaf 239.5 ^b^; Bark 200.7 ^b^; Seed 8.9 ^b^ (FL) [102]
Inca wheat (*Chenopodium quinoa*), grain	15.9 ^d^ (FL) [101]
Indian gooseberry (*Phyllanthus emblica*), fruit	86.5 ^d^ (FL) [166]
Indian madder (*Rubia cordifolia*)	640 ^d^ (FL) [91]
Japanese Pagoda Tree (*Sophora japonica*), herb	1300 ^d^ (FL) [91]
Japanese thistle (*Cirsium japonicum*)	400 ^d^ (FL) [91]
Jeriva (*Syagrus romanzoffiana*) fruit	Pulp, 169 ^d^; Peel, 146 ^d^; Seeds, 109 ^d^ (FL) [217]
Jujube, Chinese date (*Ziziphus jujuba*), fruit	105 ^d^ and 93.8 ^d^ (FL) [166], 40 ^d^ (FL) [91]
Jute mallow (*Corchorus olitorius*), leaves	130 ^d^ (FL) [103], 101.7 ^b^ (T-FL) [174]
Kaeng Kari Gai (curry soup with coconut milk, potato and chicken, Thai dish)	2.75 ^b^ (FL) [218]
Kale (*Brassica oleracea* var. *alboglabra*)	17.7 ^b^ (FL) [80], 33.1 ^b^ (FL) [175]
Kamounia, Tunisian dish	1036 ^d^ (FL) [176]
Ketchup	5.78 ^b^ (T-FL) [23]
Khaoniaodam piak phueak (Black glutinous rice pudding, Thai dish)	1.54 ^b^ (FL) [218]
Khua kling mu (stir-fried pork with herbs, Thai dish)	15.0 ^b^ (FL) [218]
Kiwifruit (*Actinidia chinensis*), raw, fresh	6.05 ^b^ (FL) [185], 8.38 ^b^ (FL) [117], 7.0 ^b^ (T-FL) [174], 8.62 ^b^ (T-FL) [164], 9.2 ^b^ (T-FL) [24]
Kiwi fruit, gold, raw	12.1 ^b^ (T-FL) [164]
Kiwi juice (market)	9.2 (FL), 12.14 (FL-EDTA) ^a^ [116]
Kombucha, traditional	1.82 ^a^ (FL) [219]
Ladybells, strict (*Adenophora stricta*), fruit	104 ^d^ (FL) [166]
Lady’s Finger (*Abelmoschus esculentus*)	25.3 ^b^ (FL) [175], 18.9 ^b^ (T-FL) [174]
Lady’s mantle (*Alchemilla glabra*), leaves	1337 ^d^ (FL) [186]
Laurel (*Laurus nobilis*), leaves	31.7 ^b^ (PE) [170], 837 ^d^ (FL) [178]
Lavender, English (*Lavandula angustifolia*)	16.2 ^b^ (PE) [170]
Leek *Allium ampeloprasum* var. *porrum*	4.90 ^b^ (FL) [98], 5.69 ^b^(FL) [164], 15.2 ^b^ (FL) [175]
Leek, Romana	9.10 ^b^ (FL) [98]
Leek, Rossa di Trento	33.2 ^b^ (FL) [98]
Leek sprouts	8.21 ^b^ (FL) [115]
Lemon (*Citrus limon*), fruit, unpeeled	13.5 ^b^ (T-FL) [164], 1043 ^d^ (FL) [220]
Lemon juice	12.3 ^b^ (FL) [164], 12.6 ^a^ (FL) [23]
Lemon balm *Melissa officinalis* leaves	9.54 ^b^ (PE) [170], 60.0 ^b^ (FL) [98], 476.6 ^d^ (FL) [101], 1121 ^d^ (FL) [178]
Lemon thyme (*Thymus* × *citriodorus*)	13.3 ^b^ (PE) [170]
Lemon verbena (*Aloysia triphylla*)	17.4 ^b^ (PE) [170], 38 ^c^ (PGR) [72]
Lentil (*Lens culinaris*), raw	72.8 ^b^ (FL) [166], 80.8 ^b^ (FL) [179], 97.7 ^b^ (FL) [180]
Lentil, red, flour	17.1 ^b^ (FL) [167]
Lettuce (*Lactuga sativa*), Catalogna	10.5 ^b^ (FL) [98]
Lettuce, Cappuccio estiva ‘Kagnran’	9.56 ^b^ (FL) [98]
Lettuce, Cocarde	21.3 ^b^ (FL) [98], 4.91 ^b^ (T-FL) [174]
Lettuce, butterhead	14.2 ^b^ (T-FL) [23]
Lettuce, green leaf	15.3 ^b^ (T-FL) [164], 15.5 ^b^ (T-FL) [23]
Lettuce, iceberg (*Lactuca sativa* var. *capitata nidus jaggeri*	2.99 ^b^ (FL) [175], 4.06 ^b^ (FL) [117, 4.51 ^b^ (T-FL) [23]
Lettuce, red leaf	10.5 ^d^ (L-FL) [135], 17.9 ^b^ (T-FL) [23], 24.3 ^b^ (T-FL) [164]
Lettuce, romaine	9.89 ^b^ (T-FL) [23]
Licorice (*Glycyrrhiza glabra*), root	212 ^b^ (FL) [182], 670 ^d^ (FL) [178]
Lime (*Tilia cordata*), flowers	1020 ^d^ (FL) [178]
Lime (*Citrus aurantifolia*), fruit, raw	0.82 ^b^ (T-FL) [164]
Lime juice	8.23 ^b^ (FL) [164], 8.56 ^a^ (FL) [23]
Longan (*Dimocarpus longan*), fruit	3.3 ^b^ (FL) [192], 75.0 ^d^ (FL) [166]
Loquat, Japanese plum (*Eriobotrya japonica*), fruit	93.5 ^d^ (FL) [166]
Lotus (*Nelumbo nuficera*) root	21.8 ^b^ (FL) [175], 77.0 ^b^ (FL) [110], 1300 ^d^ (FL) [91], 12.2 ^b^ (T-FL) [174]
Lovage (*Levisticum officinale*)	21.5 ^b^ (PE) [170], 57.3 ^b^ (FL) [181]
Love-lies-bleeding (*Amaranthus caudatus*), grain	6.5 ^d^ (FL) [101]
Lychee (*Litchi chinensis*) fruit	5.4 ^b^ (FL) [192]
Lychee fruit pulp, peeled, de-seeded	34.1 ^b^ (FL) [145]
Macadamia (*Macadamia* spp.) nuts	17.0 ^b^ (T-FL) [23,24]
Madagascar periwinkle (*Catharanthus roseus*)	22.3 ^b^ (PE) [170]
Magnolia vine (*Schisandra chinensis*), fruit	112 ^d^ (FL) [166]
Maidenhair tree (*Gingko biloba*)	13.2 ^b^ (PE) [170]
Makiang (*Cleistocalyx nervosum* var *paniala*), fruit, raw	37.0 ^b^ (FL) [177]
Maloud (*Elaeagnus latifolia*), fruit, raw	26.1 ^b^ (FL) [177]
Mamey sapote (*Pouteria sapota*) fruit	6.6 ^b^ (FL) [192]
Mango (*Mangifera indica*), fruit	2.2 ^b^ (ripe), 1.5 ^b^ (green) (FL) [192], 7.4 ^b^, 21.0 ^b^ (FL) [177], 13.0 ^b^ (FL) [164], 10.0 ^b^ (T-FL) [23],
Mangosteen (*Garcinia mangostana*), fruit, raw	25.1 ^b^ (FL) [177]
Maple (*Acer* spp.) syrup	4.34 ^a^ (FL) [107]
Maqui (*Aristotelia chilensis*) berry	198.5 ^b^ (FL) [114]
Maqui berry, concentrated powder	750 ^b^ (FL) [51]
Maqui berry pomace	10.6 ^b^ (FL) [221]
Marigold (*Calendula officinalis*), flowers	407 ^d^ (FL) [178]
Marionberry (*Rubus ursinus*)	28.0 ^b^ (PE) [187]
Marjoram (*Origanum majorana*)	273.0 ^b^ (FL) [96]
Marple (*Acer saccharum*) syrup	5.90 ^b^ (T-FL) [164]
Marrow (*Cucurbita pepo*)	2.9 ^b^ (FL) [181]
Mashua (*Tropaeolum tuberosum*) tuber	40.5 ^d^ (yellow), 260.9 ^d^ (purple) (FL) [101], 265 ^d^ and 402 ^d^ (FL) [222]
Meadowsweet (*Filipendula ulmaria*), leaves	1555 ^d^ (FL) [186]
Meatballs with sauce, French dish	451.0 ^d^ (FL) [176]
Meatballs, Königsberger, German dish	1008 ^d^ (FL) [176]
Melon (*Cucumis melo*), cantaloupe, raw	3.19 ^b^ (T-FL) [164], 2.22–3.54 ^b^ (T-FL) [174]
Melon, honeydew, raw	2.53 ^b^ (T-FL) [164]
Melon, Andesu	3.02 ^b^ (T-FL) [174]
Melon, Earl’s Favorite	1.94 ^b^ (FL) [110]
Melon, Kinsho	2.70 ^b^ (T-FL) [174]
Melon, Quincy	3.54 ^b^ (T-FL) [174]
Melon, Takami	2.22 ^b^ (T-FL) [174]
Mess apple (*Bellucia grossularioides*) fruit	26.8 ^b^ (FL) [108]
Mexican oregano (*Poliomintha longiflora*)	96.2 ^b^ (PE) [170]
Milk, cow’s, skimmed, 0.1% fat	20.9 ^a^ [97]
Milk, semi-skimmed	8.18 ^b^ (FL) [117]
Milk, 2%, chocolate-flavored	12.63 ^a^ (FL) [23]
Milk, human	1.83 ^a^ (FL) [223], 2.46 ^a^ (Winnipeg), 3.41 ^a^ (Vancouver) (FL) [224]
Mountain tea (*Sideritis scardica*), aerial parts	778 ^d^ (FL) [178]
Mtewem, Algerian dish	472.0 ^d^ (FL) [176]
Mu phat phrik khing (stir-fried pork with herbs, Thai dish)	5.42 ^b^ (FL) [218]
Mulberry, white (*Morus alba*), fruit	3001 ^d^ (FL) [208]
Mulberry, Himalayan (*Morus macroura*), fruit	92.0 ^d^ (FL) [166]
Mulukhiya (*Corchorus olitorius*)	83.5 ^b^ (FL) [110]
Murtilla (*Ugni molinae*)	107.7 ^b^ (FL) [114]
*Matricaria chamomilla* extract	431 ^c^ (FL) [72], 9 ^c^ (PGR) [72]
Mush, blue corn with ash	6.84 ^b^ (FL) [164]
Mushroom, beech mushroom (*Hypsizygus marmoreus*)	12.2 ^b^ (T-FL) [174]
Mushroom, abalone (*Pleurotus cystidiosus*), raw	16.5 ^b^ (FL) [129]
Mushroom, abalone, boiled	3.59 ^b^ (FL) [129]
Mushroom, chaga (*Inonotus obliquus*)	630 ^d^ (FL) [87]
Mushroom, crimini (*Agaricus bisporus)*	105.7 ^b^ (T-FL) [225]
Mushroom, Giant Panus (*Pleurotus levis*)	3901 ^d^ (FL) [226]
Mushroom, golden needle (*Flammulina velutipes*), raw	13.60 ^b^ (FL) [129], 6.66 ^b^ (FL) [175]
Mushroom, golden needle, boiled	4.20 ^b^ (FL) [129]
Mushroom, *Hydnellum ferrugineum*	90.7 ^b^ (FL) [132]
Mushroom, hygroscopic earthstar (*Astraeus hygrometricus*), raw	25.1 ^b^ (FL) [129]
Mushroom, hygroscopic earthstar, boiled	20.8 ^b^ (FL) [129]
Mushroom, Indian Oyster (*Pleurotus pulmonarius*)	9555, 24,662 ^d^ (FL) [226]
Mushroom, *Inonotus hispidus*	290.0 ^b^ (FL) [132]
Mushroom, jelly mushroom (*Auricularia polytricha*)	1.60 ^b^ (FL) [175]
Mushroom, Jew’s ear (*Auricularia auricula-judae*), raw	2.15 ^b^ (FL) [129]
Mushroom, Jew’s ear, boiled	0.76 ^b^ (FL) [129]
Mushroom, King Oyster mushroom (*Pleurotus eryngii*)	7.59 ^b^ (T-FL) [174], 11.7 ^b^ (FL) [129]
Mushroom, King Oyster mushroom, boiled	4.82 ^b^ (FL) [129]
Mushroom, lion’s mane (*Hericium erinaceus*)	26 ^d^ (FL) [87]
Mushroom, maitake (*Grifola frondosa*)	39.3 ^b^ (T-FL) [225], 190 ^d^ (FL) [87]
Mushroom, *Phaeolus schweinitzii*	340.0 ^b^ (FL) [132]
Mushroom, oyster mushroom (*Pleurotus ostreatus*)	8.02 ^b^ (FL) [175], 13,5 ^d^, 14,1 ^d^ (FL) [226], 90.7 ^b^ (FL) [132], 19.2 ^b^ (T-FL) [174], 55.3 ^b^ (T-FL) [225]
Mushroom *Pleurotus* sp. flour	20.8 ^d^ (FL) [227]
Mushroom, portabella *(Agaricus bisporus)*	138.3 ^b^ (T-FL) [225]
Mushroom, reishi (*Ganoderma* sp.)	23 ^d^ (FL) [87]
Mushroom, sajor-caju (*Lentinus sajor-caju*), raw	8.41 ^b^ (FL) [129]
Mushroom, sajor-caju, boiled	1.92 ^b^ (FL) [129]
Mushroom, shiitake (*Lentinula edodes*)	6.10 ^b^ (FL) [175], 16.4 ^b^ (FL) [129], 30 ^d^ (FL) [87], 62.7 ^b^ (T-FL) [225]
Mushroom, straw mushroom (*Volvariella volvacea*), raw	16.5 ^b^ (FL) [129]
Mushroom, straw mushroom, boiled	8.54 ^b^ (FL) [129]
Mushroom, tea tree mushroom (*Cyclocybe cylindracea*), raw	27.7 ^b^ (FL) [129]
Mushroom, tea tree mushroom, boiled	12.2 ^b^ (FL) [129]
Mushroom, *Tricholoma caligatum*	158.0 ^b^ (FL) [132]
Mushroom, *Tricholoma columbetta*	155.0 ^b^ (FL) [132]
Mushroom, turkey tail (*Trametes versicolor*)	36 ^d^ (FL) [87]
Mushroom, white button (*Agaricus bisporus*)	19.9 ^b^ (FL) [175], 65.8 ^b^ (FL) [132], 86.3 ^b^ (T-FL) [225]
Mushroom, white jelly fungus (*Tremella fusiformis*), raw	1.96 ^b^ (FL) [129]
Mushroom, white jelly fungus, boiled	0.91 ^b^ (FL) [129]
Mustard, white (*Sinapis alba*), fruit	69.3 ^d^ (FL) [166]
Mustard seed, yellow, ground	292.6 ^b^ (T-FL) [23]
Myoga (*Zingiber mioga*)	6.78 ^b^ (T-FL) [174]
Myrrh (*Commiphora molmol*)	96 ^d^ (FL) [91]
Nam phrik ong (curry dip with pork and herb, Thai dish)	4.57 ^b^ (FL) [218]
Nance (*Byrsonima crassifolia*)	Leaf, 778.8 ^b^; Bark, 590.8 ^b^; Fruit, 11.8 ^b^ (FL) [102]
Narrowleaf cattail (*Typha angustifolia*)	120 ^d^ (FL) [91]
Nectarine (*Prunus persica var. nucipersica*), fruit, raw	7.49 ^b^ (T-FL) [23], 9.19 ^b^ (T-FL) [164]
Nettle (*Urtica dioica*), leaves	162 ^d^ (FL) [178]
Noni (*Morinda citrifolia*) fruit, raw	8.00 ^b^ (FL) [164]
Nutmeg (*Myristica fragrans*), ground	696.4 ^b^ (T-FL) [164], 1187 ^b^ (FL) [144]
Oat (*Avena sativa*), flour bran	17.1–21.2 ^d^ (FL) [228] 19.7–25.6 ^d^ (FL) [228]
Oca (*Oxalis tuberosa*)	14.7 ^d^ (FL) [101]
Olive (*Olea europoea*), oil, extra virgin	1.06–6.20 ^b^ (PE) [84], 3.72 ^b^ (FL) [164], 11.5 ^b^ (FL) [96]
Onion (*Allium cepa*), green	14.7 ^b^ (FL) [181]
Onion, red raw	15.2 ^b^ (FL) [164], 11.5 ^b^ (T-FL) [23],12.2 ^b^ (T-FL) [174]
Onion, sweet raw	5.94 ^b^ (FL) [117], 6.14 ^b^ (T-FL) [164], 6.15 ^b^ (T-FL) [23]
Onion, yellow raw	6.70 ^b^ (FL) [110], 16.9 ^b^ (FL) [175], 62.5 ^b^ (FL) [229], 9.13 ^b^ (T-FL) [164], 10.3 ^b^ (T-FL) [23], 7.59 ^b^ (T-FL) [174]
Onion, yellow cooked	12.2 ^b^ (FL) [23]
Onion, yellow, sauteed	12.2 ^b^ (FL) [164]
Onion, white raw	8.63 ^b^ (FL) [164]
Onion, Bianca di maggio	3.42 ^b^ (FL) [98]
Onion, Rossa di tropea	15.2 ^b^ (FL) [98]
Onion powder	42.9 ^b^ (T-FL) [164], 57.4 ^b^ (T-FL) [23]
Onion, spring (*Allium fistulosum*)	13.0 ^b^ (FL) [175]
Orange (*Citrus* × *sinensis*)	12.7 ^b^ and 19.0 ^b^ (FL) [211]
Orange, Navel	18.1 ^b^ (T-FL) [24], 18.2 ^b^(T-FL) [164]
Orange juice	7.26 ^b^ (FL) [21], 7.64 ^b^ (FL) [117], 10.8 ^a^ (FL) [21], 11.38 ^a^ (FL), 16.02 ^a^ (FL-EDTA) [116], 1.3–21.6 ^a^ (PGR) [134]
Orange juice, canned	7.03 ^b^ (FL) [164]
Orange, peel	510 ^b^ (FL) [182]
Orange mint (*Mentha aquatica*)	18.8 ^b^ (PE) [170]
Oregano (*Origanum vulgare*) fresh	139.7 ^b^ (FL) [98]
Oregano leaf, dried	1233 ^b^ (FL) [144], 1233 ^b^ and 2001.3 ^b^ (T-FL) [23], 1753 ^b^ (T-FL) [164]
Oregano essential oil	9.03 ^b^ (FL) [130]
Oregano, Greek mountain (*Origanum vulgare* ssp. *hirtum*)	64.7 ^b^ (PE) [170]
Oregano, Cuban (*Plectranthus amboinicus*)	4.71 ^b^ (PE) [170]
Oriental arborvitae (*Platycladus orientalis*)	940 ^d^ (FL) [91]
Paella, Valencian (Spanish dish)	778.0 ^b^ (FL) [176]
Papaya (*Carica papaya*), fruit, raw	3.0 ^b^ (FL) [175], 3.00 ^b^(T-FL) [164]
Papaya, cv. Red Lady	2.6 ^b^ (green), 5.3 ^b^ (ripe) (FL) [192]
Paprika (*Capsicum annuum*)	179.2 ^b^ (T-FL) [23], 219.3 ^b^ (T-FL) [164]
Paprika, smoked	192.1 ^b^ (FL) [117]
Parsley (*Petroselium hortense*) raw	11.0 ^b^ (PE) [170], 13.0 ^b^ (FL) [98]
Parsley, dried	736.7 ^b^ (FL) [51], 743.5 ^b^ (T-FL) [23]
Parsley leaves	108.6 ^b^ (FL) [181]
Passion fruit (*Passiflora mollissima*), fruit	205.0 ^d^ (FL) [101]
Patawa (*Oenocarpus bataua*), fruit, peeled	1627 ^d^ (FL) [163]
Pea soup, German dish	560.8 ^d^ (FL) [176]
Peach (*Persica vulgaris*), raw	6.2 ^b^ (FL) [101], 15.9 ^b^ (FL) [115], 18.7 ^b^ (FL) [117], 85 ^d^ (FL) [91], 18.6 ^b^ (T-FL) [23], 19.2 ^b^ (T-FL) [164]
Peach, E. Lady, unpeeled	31.3 ^b^ (FL) [114]
Peach, E. Lady, peeled	19.5 ^b^ (FL) [114]
Peach, Zee Lady, unpeeled	52.1 ^b^ (FL) [114]
Peach, Zee Lady, peeled	15.8 ^b^ (FL) [114]
Peach canned, heavy syrup	4.19 ^b^ (FL) [23], 4.36 ^b^ (FL) [164]
Peach juice	16.0 ^a^ (PGR) [134]
Peanuts (*Arachis hypogaea*)	31.7 ^b^ (T-FL) [23]
Peanut, raw-blanched	30.4 ^b^ (T-FL) [230]
Peanut, roasted-blanched	34.6 ^b^ (T-FL) [230]
Peanut flour	59.1–79.9 ^b^ (T-FL) [230]
Peanut butter	34.3 ^b^ (T-FL) [23]
Peanut seed oil	1.1 ^b^ (FL) [98]
Pear (*Pyrus* spp.)	29.4 ^b^ (FL) [115]
Pear, green cultivars, with peel, raw	19.1 ^b^ (T-FL) [23], 21.5 ^b^ (FL) [117], 22.0 ^b^ (T-FL) [164]
Pear, Abate, peel	83.6 ^b^ (FL) [115]
Pear, Abate, pulp	9.21 ^b^ (FL) [115]
Pear, B. Bosc, unpeeled	29.0 ^b^ (FL) [114]
Pear, B. Bosc, peeled	15.8 ^b^ (FL) [114]
Pear, Conference, peel	57.0 ^b^ (FL) [115]
Pear, Conference, pulp	11.7 ^b^ (FL) [115]
Pear, Red Anjou	17.5 ^b^ (T-FL) [164], 17.7 ^b^ (T-FL) [23]
Pear juice	7.04 ^b^ (FL) [164]
Pea (*Pisum sativum*), split, mature seeds, raw	5.24 ^b^ (FL) [164], 96.8 ^b^ (T-FL) [174]
Pea, blackeye, dried	43.4 ^b^ (T-FL) [23]
Pea, green	5.90 ^b^ (FL) [179], 40.3 ^b^ (FL) [180]
Pea, green, canned	3.84 ^b^ (T-FL) [23]
Pea, green, frozen	6.00 ^b^ (T-FL) [23]
Pea, yellow raw	7.41 ^b^ (FL) [164], 8.35 ^b^ (FL) [179], 33.4 ^b^ (FL) [180]
Pea flour	10.3 ^d^ (FL) [126]
Pecan (*Carya illinoinensis*)	179.4 ^b^ (T-FL) [23,24]
Peony (*Paeonia officinalis*), petals	555.2 ^b^ (FL) [171]
Pepper (*Capsicum annum* var. *grossum*), green	5.35 ^b^ (FL) [175], 5.6 ^b^ (FL) [181], 10.6 ^b^ (FL) [98]
Pepper, green sweet, raw	5.58 ^b^ (T-FL) [23], 9.35 ^b^ (T-FL) [164]
Pepper, green sweet, cooked	6.15 ^b^ (FL) [23]
Pepper, orange sweet raw	9.84 ^b^ (T-FL) [23]
Pepper, red	8.42 ^b^ (FL) [98], 9.3 ^b^ (FL) [181], 12.7 ^b^ (FL) [175], 138 ^d^ (FL) [166], 196.7 ^b^ (T-FL) [164]
Pepper, red sweet, raw	8.21 ^b^ (T-FL) [164], 9.01 ^b^ (T-FL) [23]
Pepper, red sweet, cooked	8.47 ^b^ (FL) [23]
Pepper, yellow, raw	9.50 ^b^ (FL) [115], 9.50 ^b^ (FL) [98], 10.2 ^b^ (T-FL) [23], 10.4 ^b^ (T-FL) [164]
Pepper, yellow, grilled	6.94 ^b^ (FL) [115]
Pepper, white	407.0 ^b^ (T-FL) [164]
Peppercorn, black (*Piper nigrum*)	395 ^b^ (FL) [144], 301.4 ^b^ (T-FL) [23]
Peppercorn, black, ground	250.99 ^b^ (T-FL) [23], 340.5 ^b^ (T-FL) [164]
Peppermint (*Mentha piperita*), fresh	139.8 ^b^ (FL) [98],15.8 ^b^ (PE) [170]
Peppermint, leaves	2917 ^d^ (FL) [178], 15.8 ^b^ (PE) [170]
Perilla (beefsteak plant) (*Perilla frutescens*), leaf	107 ^d^ (FL) [166], 286.2 ^b^ (T-FL) [174]
Perilla, green	318.0 ^b^ (T-FL) [174]
Perilla, red	262.2 ^b^ (T-FL) [174]
Peruvian elderberry (*Sambucus peruviana*)	361.3 ^d^ (FL) [101]
Peruvian ground apple (*Smallanthus sonchifolius*), root	134.0 ^d^ (FL) [101]
Pine (*Pinus pinea*) nuts, dried	7.19 ^b^ (T-FL) [23]
Pineapple (*Ananas comosus*)	3.85 ^b^ (T-FL) [164], 7.93 ^b^ (T-FL) [23]
Pineapple, extra sweet variety, raw,	9.43 ^b^ (T-FL) [164]
Pineapple, traditional varieties, raw	5.62 ^b^ (T-FL) [164]
Pineapple juice	5.68 ^b^ (FL) [164], 15.2 ^a^ (PGR) [134]
Pineapple juice, canned	5.68 ^b^ (FL) [164]
Pineapple sage (*Salvia elegans*)	11.6 ^b^ (PE) [170]
Pink arnebia (*Arnebia euchroma*)	320 ^d^ (FL) [91]
Pistachio (*Pistacia vera*) nuts	76.8 ^b^ (T-FL) [164], 79.8 ^b^ (T-FL) [23,24]
Pitaya (*Hylocereus* spp.)	7.6 ^b^ (red), 3.0 ^b^ (white) (FL) [192]
Plantain, broadleaf (*Plantago major*), leaf	620.9 ^d^ (FL) [101]
Plantain, Asian (*Plantago asiatica*), leaves	93.7 ^d^ (FL) [166]
Plum (*Prunus domestica*), raw	9.49 ^b^ (FL) [185], 10.8 ^b^ (FL) [101], 61.0 ^b^ (T-FL) [164], 62.4 ^b^(T-FL) [24]
Plum, Black Diamond, with peel, raw	75.8 ^b^ (FL) [51], 73.4 ^b^ (T-FL) [23]
Plum, Blackam, unpeeled	48.6 ^b^ (FL) [114]
Plum, Blackam, peeled	31.6 ^b^ (FL) [114]
Plum, Romanian summer varieties	11.8–23.7 ^b^ (FL) [127]
Plum, Romanian autumn varieties	15.1–34.4 ^b^ (FL) [127]
Plum tart, Luxembourg	188.5 ^d^ (FL) [176]
Pomegranate (*Punica granatum*), raw	19.7 ^b^ (FL) [101], 44.8 ^b^ (FL) [164], 14.3 ^b^ (T-FL) [174]
Pomegranate, peel	149 ^d^ (FL) [166], 192.7–237.2 ^b^ (FL) [231]
Pomegranate, juice	5.94–8.18 ^a^ (FL) [231]
Pomegranate juice, bottled	26.8 ^b^ (FL) [164]
Popcorn, air-popped	17.4 ^b^ (T-FL) [164]
Popcorn, buttered, premium	17.4 ^b^ (T-FL) [23]
Poppy (*Papaver rhoeas*), seed	4.80 ^b^ (T-FL) [23], 4.81 ^b^ (T-FL) [164]
Potato (*Solanum tuberosum*)	5.07 ^b^ (FL) [110], 6.46 ^b^ (FL) [175], 10.3 ^b^ (FL) [181], 33.1–346.5 ^b^ (FL) [100], 6.28 ^b^ (T-FL) [174]
Potato, purple (‘Purple Majesty’), flour	74.6 ^d^ (FL) [126]
Potato, russet, raw	16.8 ^b^ (FL) [51], 13.2 ^b^ (T-FL) [23]
Potato, russet, cooked	15.6 ^b^ (T-FL) [23], 16.8 ^b^ (T-FL) [164]
Potato, red, raw	9.05 ^b^ (T-FL) [174], 11.0 ^b^ (T-FL) [23]
Potatoes, red, cooked	13.3 ^b^ (T-FL) [23]
Potatoes, white, raw	10.6 ^b^ (T-FL) [23]
Potatoes, white, cooked	10.8 ^b^ (T-FL) [23], 11.4 ^b^ (T-FL) [164]
Potato, 10 varieties grown in Peru, raw	57.0–357.8 ^d^ (FL) [232]
Potato omelet, Spain	96.5 ^d^ (FL) [176]
Prickly pear cactus (*Opuntia ficus indica*) fruit	45.6 ^d^ (FL) [101]
Prune juice, canned	20.4 ^b^ (FL) [21]
Pummelo x grapefruit hybrid (*Citrus paradisi* var Jaffa Sweetie)	16.7 ^b^ (FL) [211]
Pumpkin (*Cucurbita pepo*), raw	4.9 ^b^ (FL) [101], 4.83 ^b^ (T-FL) [23], 8.44 ^d^ (L-FL) [135]
Pumpkin (*Cucurbita maxima*)	9.58 ^b^ (FL) [110], 8.75 ^b^ (T-FL) [174]
Pumpkin, tropical (*Cucurbita moschata*)	3.91 ^b^ (FL) [175]
Puncturevine, caltrop (*Tribulus terrestris*), fruit	95.4 ^d^ (FL) [166]
Purple osier willow (*Salix sinopurpurea*), fruit	77.4 ^d^ (FL) [166]
Quinoa (*Chenopodium quinoa*), black, flour	32.4 ^b^ (FL) [167]
Quinoa red, flour	27.5 ^b^ (FL) [167]
Quince, unpeeled	60.1 ^b^ (FL) [114]
Quince, peeled	42.9 ^b^ (FL) [114]
Radish *Raphanus sativus*, raw	5.82 ^b^ (FL) [175], 23.6 ^b^ (FL) [181], 9.54 ^b^ (T-FL) [23,24], 17.5 ^b^ (T-FL) [164], 21.8 ^b^ and 25.1 ^b^ (T-FL) [174]
Radish, Jolly	12.4 ^b^ (FL) [98]
Radish, Tondo	36.0 ^b^ (FL) [98]
Radish, leaves	24.7 ^b^ and 47.9 ^b^ (T-FL) [174]
Radish, sango sprouts	35.8 ^b^ (FL) [115]
Radish sprouts	21.8 ^b^ (FL) [115]
Raisins	28.3 ^b^ (FL) [185], 30.4 ^b^ (T-FL) [23,24]
Raisin, golden	104.55 ^b^ (FL) [233]
Raisin, seedless	34.1 ^b^ (T-FL) [164]
Raisin, sun-dried	37.4 ^b^ (FL) [233]
Raisin, white	9.30 ^b^ (FL) [115]
Ramie (*Bochmeria nivea*)	640 ^d^ (FL) [91]
Rapeseed (*Brassica napus* subsp. *napus*) oil, pressed, crude oil, extracted, crude oil, extracted, bleached	6.82 ^b^ and 6.40 ^b^ (FL) [109] 9.94 ^b^ and 11.1 ^b^ (FL) [109] 3.02 ^b^ (FL) [109]
Raspberry, European red (*Rubus idaeus*)	24.0 ^b^ (PE) [187], 5.5 ^d^ (FL) [184], 28.7 ^b^ (FL) and 38.9 ^b^ (FL) [101], 50.7 ^b^ (FL) [51], 129 ^d^ (FL) [166], 161 ^d^ (FL) [162], 49.3 ^b^ (T-FL) [24], 14.5 ^b^ (PGR) [73]
Raspberry, leaves	1349 ^d^ (FL) [186]
Raspberry juice	23.1 ^a^ (PE), 54.0 ^a^ (FL) [19]
Raspberry pomace	10.8 ^b^ (FL) [221]
Raspberry, black (*Rubus occidentalis*)	77.2 ^b^ (PE) [187], 192.2 ^b^ (FL) [164]
Raspberry, boysenberry (*R. ursinus × idaeus*)	42.2 ^b^ (PE) [187]
Raspberry, evergreen (*Rubus laciniatus*)	27.5 ^b^ (PE) [187]
Rechta, Algeria	427.8 ^d^ (FL) [176]
Red chicory (*Cichorium intybus*)), fresh	35.4 ^b^ (FL) [115]
Red chicory, cooked	37.8 ^b^ (FL) [115]
Rhubarb (*Rheum rhaponticum*)	13.2 ^b^ (T-FL) [176]
Rice (*Oryza sativa*), brown	14.4 ^b^ and 17.5 ^b^ (T-FL) [174]
Rice bran, crude	242.9 ^b^ (T-FL) [23,24]
Rice, Ermes, flour	14.4 ^b^ (FL) [167]
Rice, Nerone, flour	72.3 ^b^ (FL) [167]
Rice, orange, flour	25.5 ^b^ (FL) [167]
Rice, wild, flour	31.8 ^b^ (FL) [167]
Rice, black, flour	39.6 ^b^ (FL) [167]
Rice, violet, flour	117.8 ^b^ (FL) [167]
Rice beverage	1.3 ^a^ (FL) [193]
Rice pudding with sugar and cinnamon, German dish	571.3 ^d^ (FL) [176]
Rocket (arugula, *Eruca vesicaria*), raw	19.0 ^b^(FL) [164], 23.3 ^b^ (FL) [115], 23.7 ^b^ (FL) [98], 2319.0 ^b^ (FL) [164], 32.7 ^b^ (T-FL) [174]
Rocket, cooked	10.1 ^b^ (FL) [115]
Rosehip (*Rosa canina*)	961.5 ^b^ (T-FL) [164], 1085 ^b^ (FL) [144]
Roselle (*Hibiscus sabdariffa*), leaves	340 ^d^ (FL) [103]
Rosemary (*Rosmarinus officinalis*)	19.2 ^b^ (PE) [170], 2.90 ^b^ (FL) [98]
Rosemary dried	1652.8 ^b^ (T-FL) [164]
Rosemary extract	14,300 ^b^ and 18,200 ^b^ (FL) [18]
Rowanberry (*Sorbus aucuparia*)	80.9 ^b^ (FL) [101]
*Rubus hirsutus*	5.7 ^d^ (FL) [184]
*Rubus microphyllus*	8.1 ^d^ (FL) [184]
*Rubus palmatus*	5.6 ^d^ (FL) [184]
*Rubus trifidus*	3.0 ^d^ (FL) [184]
*Rubus* × *medius*	4.9 ^d^ (FL) [184]
Russian olive (*Elaeagnus angustifolia*), fruit	90.6 ^d^ (FL) [166]
Sacha inchi (*Plukenetia volubilis*), seeds	6.5–9.8 ^b^ (T-FL) [101]
Safflower (*Carthamus tinctorius*)	370 ^d^ (FL) [91]
Sage (*Salvia officinalis*)	13.3 ^b^ (PE) [168], 320 ^b^ (FL) [98]
Sage, ground	1199.3 ^b^ (T-FL) [164]
Sage, leaves	966 ^d^ (FL) [178]
Saint John’s wort (*Hypericum perforatum*), aerial parts	16.8 ^b^ (PE) [170], 1141 ^d^ (FL) [178]
Salad burnet (*Sanguisorba minor*)	8.33 ^b^ (PE) [170]
Salsa sauce	10.0 ^b^ (T-FL) [23]
Saltwort (*Salsola komarovii*)	45.9 ^b^ (T-FL) [174]
Sardine meatballs, Italian dish	823.7 ^d^ (FL) [176]
Savory *Santureja hortensis*	96.5 ^b^ (FL) [98]
Sea buckthorn (*Hippophae rhamnoides*), fruit	168 ^d^ (FL) [166]
Seagrass (*Posidonia oceanica*) leaves	104 ^d^ (FL) [234]
Seaweed (*Porphyra yezoensis*)	61.1 ^d^ (FL) [175]
Seriguela (*Spondias purpurea*), fruit, pulp	44.2 ^d^ (FL) [235]
Seriguela, fruit, peel	31.0 ^d^ (FL) [235]
Seriguela, seed	46.8 ^d^ (FL) [235]
Sesame (*Sesamum indicum*) seed, black	78.4 ^d^ (FL) [236]
Sesame seed, white	37.6 ^d^ FL) [236]
Shiny bugleweed (*Lycopus lucidus*)	1220 ^d^ (FL) [91]
Sichuan pepper (*Zanthoxylum* spp.)	1184.0 ^b^ (T-FL) [164]
Sicilian caponata (dish)	404.8 ^d^ (FL) [176]
Simple-stem bur-reed (*Sparganium stoloniferum*)	260 ^d^ (FL) [91]
Small red bean	86.1 ^b^ (FL) [51]
Snack bar, chewy low-fat granola, Quaker	16.7 ^b^ (T-FL) [23]
Snack bar, fruit and oatmeal, strawberry, Quaker	19.6 ^b^ (T-FL) [23]
Society garlic (*Tulbaghia violacea*)	7.50 ^b^ (PE) [170]
Sorghum (*Sorghum bicolor*), grain, white	22.0 ^b^ (T-FL) [237]
Sorghum, grain, red	140.0 ^b^ (T-FL) [237]
Sorghum, grain, black	219.0 ^b^ (T-FL) [237]
Sorghum grain, hi-tannin	454.0 ^b^ (T-FL) [51]
Sorghum, bran, white	64.0 ^b^ (T-FL) [237]
Sorghum, white, flour	22.4 ^b^ (FL) [167]
Sorghum, bran, red	710 ^b^ (FL) [51], 704 ^b^ (T-FL) [237]
Sorghum, red, flour	47.3 ^b^ (FL) [167]
Sorghum, bran, black	1008.0 ^b^ (FL) [51], 2400 ^b^ (FL) [51]
Sorghum, bran, hi-tannin	2400.0 ^b^ (FL) [51]
Sour cherry (*Prunus cerasus*), fruit	58.6 ^b^ (FL) [101]
Sour cherry, cv. Marasca	128.7 ^b^ (FL) [96]
Sour cherry, cv. Cigancica	75.2 ^b^ (FL) [96]
Soybean *Glycine max*, mature seeds, raw	54.1 ^b^(FL) [164]
Soybean, black	131.3 ^b^ (FL) [179], 162.5 ^b^ (FL) [180]
Soybean, yellow	38.7 ^b^ (FL) [179], 86.8 ^b^ (FL) [180]
Soybean sprout	9.62 ^b^ (FL) [115],15.7 ^b^ (T-FL) [174], 31.3 ^b^ (FL) [175]
Spearmint (*Mentha spicata*), leaves	8.10 ^b^ (PE) [170], 748 ^d^ (FL) [178],
Spinach *Spinacia oleracea*, raw	12.1 ^b^ (FL) [172],12.6 ^b^ (FL) [80], 15.1 ^b^(FL) [164], 16.1 ^b^ (FL) [175] (FL) [115], 27.3 ^b^ (FL) [98], 26.4 ^b^ (T-FL) [23], 27.3 ^b^ and 71.0 ^d^ (L-FL) [135]
Spinach, Ceylon (*Basella rubra*)	35.5 ^b^ (FL) [175]
Spinach, frozen	16.9 ^b^ (FL) [115]
Spinach, leaves	190 ^d^ (FL) [103]
Spinach, Chinese (*Amaranthus tricolor*)	9.66 ^b^ (FL) [175]
Squash (*Cucurbita pepo*)	3.96 ^b^ (FL) [98], 9.34 ^b^ (FL) [98]
*Stachys geobombycis*, root	125 ^d^ (FL) [166]
Stevia (*Stevia rebaudiana*) leaves	222.6 ^d^ (FL) [238]
Star anise (*Illicium verum*), fruit	136 ^d^ (FL) [166]
Strawberry (*Fragaria* × *ananassia*)	11.7 ^b^ (FL) [171], 35.8 ^b^ (T-FL) [24], 43.0 ^b^ (FL) [51], 47.2 ^b^ (FL) [101], 356 ^d^ (FL) [162], 445.1 ^b^ (T-FL) [24]
Strawberry, cv. Maya	47.1 ^b^ (FL) [96]
Strawberry, cv. Queen Elisa	49.4 ^b^ (FL) [96]
Strawberry extract	53,900 ^b^ and 54,200 ^b^ (FL) [18]
Strawberry juice	10.7 ^a^ (FL) [21], 10.7 ^a^ (FL) [21]
Sumac (*Rhus* spp.), bran, raw	3124.0 ^b^ (FL) [51]
Sumac, grain, raw	868.0 ^b^ (FL) [51]
Surinam cherry (*Eugenia uniflora*), fruit	228.0–823.4 ^d^ (FL) [124]
Sweet basil (*Ocimum basilicum*)	14.3 ^b^ (PE) [170]
Sweet granadilla (*Passiflora ligularis*) fruit	50.3 ^d^ (FL) [101]
Sweet potato (*Ipomoea batatas*), raw	6.77 ^b^ (FL) [110], 9.02 ^b^(T-FL) [164], 16.4 ^b^ (FL) [175], 8.72 ^b^ (T-FL) [174]
Sweet potato, cooked, without skin	7.66 ^b^ (T-FL) [23]
Sweet potato, dark purple-fleshed clones	14.7–29.2 ^b^ [239]
Sweet potato, orange-fleshed clones	5.89–10.3 ^b^ [239], 9.02 ^b^ (T-FL) [23]
Sweet potato, white- and yellow-fleshed clones	2.72–3.33 ^b^ [239]
Szechuan lovage (*Ligusticum chuanxiong*)	130 ^d^ (FL) [91]
Tagine with quinces and honey, Moroccan dish	918.3 ^d^ (FL) [176]
Tangerine (*Citrus tangerina*)	16.2 ^b^ (T-FL) [23]
Tangut Nitre-bush (desert cherry) (*Nitraria tangutorum*), leaves	131 ^d^ (FL) [166]
Taro (*Colocasia esculenta*)	13.0 ^b^ (FL) [175]
Tarragon (*Artemisia dracunculus*), fresh	155.4 ^b^ (FL) [98]
Tarte tatin with vanilla ice cream, Luxembourg dish	1286 ^d^ (FL) [176]
Tea (*Camellia sinensis*), black	8.71 ^a^ (PE) [19], 3.13 ^b^ (FL) [164], 17.3 ^a^ [19],1566–1629 ^d^ (FL) [19], 52.90 (PGR) [40]
Tea, black	728–1372 ^d^ (PE) [86]
Tea, black, decaffeinated	507–618 ^d^ (PE) [86]
Tea, ceremonial matcha	2142.0 ^d^ (FL) [93]
Tea, culinary matcha	2399.1 ^d^ (FL) [93]
Tea, green	632.4 ^d^ (FL) [93]
Tea, green	1239–1686 ^d^ (PE) [86]
Tea, green, brewed	12.53 ^b^ (FL) [164]
Tea, green, decaffeinated	765–845 ^d^ (PE) [86]
Tea, green, ready-to-drink	5.20 ^b^ (FL) [164]
Tea, iced	0.6–6.7 ^a^ (PGR) [134]
Tea, white	1721 ^c^ (FL) [72], 293 ^c^ (PGR)
Tea, white, ready-to-drink	2.64 ^b^ (FL) [164]
Tea, London English Breakfast	1089 ^d^ (FL), 1089 ^d^ (L-FL) [240]
Tea, Taylors Earl Gray	1134 ^d^ (FL), 1604 ^d^ (L-FL) [240]
Tea, Twinings English Breakfast	1377 ^d^ (FL), 1539 ^d^ (L-FL) [240]
Tea, Twinings Oolong	934.0 ^d^ (FL), 1303 ^d^ (L-FL) [240]
Tea, Two Leaves High Chai	653.2 ^d^ (FL), 784.9 ^d^ (L-FL) [240]
Teff (*Eragrostis tef*), flour	26.6 ^b^ (FL) [167]
Thai yam, ube (*Dioscorea alata*) tuber	24.5 ^b^ (FL) [241]
Thyme (*Thymus vulgaris*), fresh	19.5 ^b^ (PE) [170], 274.3 ^b^ (FL) [98]
Thyme, dried	1637 ^d^ (FL) [178], 1573.8 ^b^ (T-FL) [164]
Thyme, creeping (*Thymus praecox* ssp. *arcticus*)	13.4 ^b^ (PE) [170]
Tielle sétoise, French dish	307.4 ^d^ (FL) [176]
Tienchi (*Panax notoginseng*)	75 ^d^ (FL) [91]
Tiger nut (*Cyperus esculentus*) beverage	1.8 ^a^ (FL) [193]
Tiramisú, Italian dessert	754.4 ^d^ (FL) [176]
Tomato (*Solanum lycopersicum*) raw	3.89 ^b^ (FL) [175], 5.2 ^b^ (FL) [172], 5.4 ^b^ (FL) [181], 3.37 ^b^ (T-FL) [23,24], 4.18 ^b^ (T-FL) [174]
Tomato, red, rip	3.64 ^b^ (FL) [117]
Tomato, S. Marzano	6.97 ^b^ (FL) [98]
Tomato, Sarom	3.95 ^b^ (FL) [98]
Tomato, cooked	4.60 ^b^ (T-FL) [23]
Tomato, plum	5.46 ^b^ (FL) [164], 4.23 ^b^ (T-FL) [164]
Tomato juice (canned)	4.86 ^b^ (FL) [164]
Tomato juice (market)	6.47 ^a^ (FL) [23] ^a^ and 7.03 ^a^ (FL), 7.62 ^a^ (FL-EDTA) [116]
Tomato sauce	6.94 ^b^ (T-FL) [23]
Tree peony (*Paeonia suffruticosa*)	470 ^d^ (FL) [91]
Tunisian lentil soup	1015 ^d^ (FL) [176]
Turmeric (*Curcuma longa*), ground	1270.7 ^b^ (FL) [51], 1592.8 ^b^ (T-FL) [23]
Turnip (*Brassica rapa*)	1.81 ^b^ (FL) [108], 4.6 ^b^ and 11.0 ^b^ (T-FL) [174]
Turnip, leaves	11.8 ^b^ (T-FL) [174]
Ubaia (*Eugenia patrisii*)	Fruit 32.3 ^b^ (FL) Leaf 354 ^b^ (FL) [109]
Uvaia (*Eugenia pyriformis*) fruit	2.27–8.67 ^b^ (FL) [120]
Valencian hake (Spanish dish)	1010.7 ^d^ (FL) [176]
Valencian titaina (Spanish dish)	171.2 ^d^ (FL) [176]
Valerian (*Valeriana officinalis*)	15.8 ^b^ (PE) [170]
Valerian (*Valerianella locusta*), raw	22.6 ^b^ (FL) [115]
Valerian, cooked	18.2 ^b^ (FL) [115]
Vanilla (*Vanilla planifolia*) beans, dried	1224.0 ^b^ (T-FL) [164]
Vanilla extract (1g vanilla bean/25 mL 70% ethanol)	43.5 ^a^ (FL) [138]
Vietnamese coriander (*Polygonum odoratum*)	22.3 ^b^ (PE) [170]
Vinegar, apple	5.6 ^b^ (FL) [98]
Vinegar, apple and honey	2.7 ^b^ (FL) [98]
Vinegar, honey	2.3 ^b^ (FL) [98]
Vinegar, red wine	4.10 ^b^ (FL) [98]
Wajulew’s fritillary (*Fritillaria wajulewii*), fruit	81.0 ^d^ (FL) [166]
Walnut (*Juglans regia*)	135.4 ^b^ (T-FL) [23]
Walnut, defatted	235 ^b^ (FL) [242]
Walnut shells	4600–5500 ^b^ (FL) [213]
Water chestnut (*Eleocharis dulcis*)	2.26 ^b^ (FL) [175]
Water cress (*Nasturtium officinale*)	28.7 ^b^ (FL) [175]
Water spinach (*Ipomoea aquatica*)	36.9 ^b^ (FL) [175], 39.0 ^b^ and 68.5 ^b^ (T-FL) [174]
Watermelon (*Citrullus lanatus*)	13.8 ^b^ (FL) [101], 42 ^b^ (T-FL) [23]
Welsh onion (*Allium fistulosum*), blanched	4.23 ^b^ (T-FL) [174]
Welsh onion, leaves	7.00 ^b^ (T-FL) [174]
Wenyujin turmeric (*Curcuma wenyujin*)	140 ^d^ (FL) [91]
Whey, sweet	20.5 mmol TE/g protein (FL) [243]
Wine, white	3.90 (PE) [40], 1.20 (PC) [155], 3.92 ^b^ (FL) [164]
Wine, red	42.4 (PE) [40], 11. 0 (PC) [155], 31.4 ^b^ (FL) [117]
Wine, red, Burgundy	89.0 ^a^ (FL) [244]
Wine, Cabernet Sauvignon	50.3 ^b^(FL) [245], 21.2–31.7 ^a^ (PGR) [134]
Wine, red, Carmeneré	18.0 ^a^ (FL) [138]
Wine, red, Feteasca Neagra	90.1 ^a^ (FL) [244]
Wine, red, Merlot	20.2 ^a^ (FL) [138], 88.3 ^a^ (FL) [244]
Wine, red, Pinot	87.3 ^a^ (FL) [244]
Wine, red, Zinfandel	24 ^b^ (FL) [164]
Wine, table, rose	10.1 ^b^ (FL) [245]
Wine, white, Sauvignon Blanc	3.3–6.9 ^a^ (PGR) [134]
Winter savory (*Satureja montana*)	0.79 ^b^ (PE) [170]
Wormwood, sweet (*Artemisia annua*)	15.7 ^b^ (PE) [170]
Yacon (*Samallanthus sonchifolius*)	6.19 ^b^ (FL) [110]
Yam bean (*Pachyrrhizus erosus*)	2.60 ^b^ (FL) [175]
Yanhusuo (*Corydalis yanhusuo*)	130 ^d^ (FL) [91]
Yarrow (*Achillea millefolium*), flowers	842 ^d^ (FL) [178]
Zucchini (*Cucurbita pepo*), yellow	3.02 ^b^ (T-FL) [174]
Zucchini, green	4.70 ^b^ (T-FL) [174]

^a^ [mmol TE/L]; ^b^ [mmol TE/kg]; ^c^ [mg gallic acid equivalents/L]; ^d^ [mmol TE/kg dry mass]; FL, fluorescein; L, lipophilic assay; PC, *β*-phycocyanin; PE, *β*-phycoerythrin; PGR, Pyrogallol Red; T, sum of hydrophilic and lipophilic assays.

**Table 6:** Conditions of ORAC-like assays.

Assay	Conditions	Reference
HORAC	62 nM FL, 27.5 mM H_2_O_2_, 230 µM Co(II) fluoride, 75 mM phosphate buffer, pH 7.4, 37 °C	[20]
NORAC	5 µM DHR123, 50 mM Na-phosphate (pH 7.4) with 5 mM KCl and 100 µM DTPA, purged with nitrogen, 10 µM SIN-1 or 10 µM ONOO^−^ in 0.3 M NaOH, 485/530 nm	[263]
SORAC	74.1 µM xanthine/2.39 µM DHE, 150 µL/15.6 mU/mL XO, 75 mM phosphate buffer, pH 7.4, with 100 µM DTPA; control with SOD to ensure specificity	[264]
SOAC	25 µM DHE 125 µL, 0.33 mM Na_2_MoO_4_, 1.64 mM H_2_O_2_ in N,N-dimethylacetamide, 530/620 nm	[263]

DHE, dihydroethidium; DHR123, dihydrorhodamine 123; DTPA, diethylenetriaminepentaacetic acid; SOD, superoxide dismutase; XO, xanthine oxidase.

## Data Availability

No new data were created or analyzed in this study.

## Abbreviations

The following abbreviations are used in this manuscript:

AAPH	2,2′-azobis(2-methylpropionamidine) dihydrochloride
AIPH	2,2′-azobis[2-(2-imidazolin-2-yl)propane]
AMVN	2,2′-azobis (2,4-dimethylvaleronitrile)
AUC	area under the curve
BDE	bond dissociation energy
BODIPY 581/591 C11	4,4-difluoro-5-(4-phenyl-1,3-butadienyl)-4-bora-3a,4a-diaza-s-indacene-3-undecanoic acid
6-CFL	6-carboxyfluorescein
DHE	dihydroethidium
DHR123	dihydrorhodamine 123
DCDHFL	2′,7′-dichlorodihydrofluorescein
DOPC	dioleoylphosphatidylcholine
DTPA	diethylenetriaminepentaacetic acid
*k_d_*	decomposition rate constant
FL	fluorescein
HORAC	Hydroxyl Radical Absorbance Capacity
L-ORAC	Lipophilic ORAC
Meo-AMNV	2,2′-azobis(4-methoxy-2,4-dimethylvaleronitrile)
NORAC	Peroxynitrite Absorbance Capacity
ORAC	Oxygen Radical Absorption Capacity
ORAC-BODIPY	ORAC employing 581/591 C11 as the probe
ORAC-DCDHFL	ORAC employing DCDHFL as the probe
ORAC-ERR	ORAC based on the EPR technique
ORAC-FL	ORAC employing fluorescein as the probe
ORAC-PC	ORAC employing phycocyanin as the probe
ORAC-PE	ORAC employing phycoerythrin as the probe
ORAC-PGR	ORAC employing Pyrogallol Red as the probe
ORAC-PYR	ORAC employing pyranine as the probe
PC	phosphatidylcholine
PE	phosphatidylethanolamine
PGR	Pyrogallol Red
R_i_	rate of generation of peroxyl radicals
RMCD	randomly methylated cyclodextrins
SOAC	Singlet Oxygen Absorbance Capacity
SORAC	Superoxide Anion Absorbance Capacity
TAC	Total Antioxidant Capacity
